# Plasmonic Fluorescence Sensors in Diagnosis of Infectious Diseases

**DOI:** 10.3390/bios14030130

**Published:** 2024-03-02

**Authors:** Juiena Hasan, Sangho Bok

**Affiliations:** Department of Electrical and Computer Engineering, Ritchie School of Engineering and Computer Science, University of Denver, Denver, CO 80208, USA; juiena.hasan@du.edu

**Keywords:** plasmonics, SPR, LSPR, infectious disease, biosensors, fluorescence

## Abstract

The increasing demand for rapid, cost-effective, and reliable diagnostic tools in personalized and point-of-care medicine is driving scientists to enhance existing technology platforms and develop new methods for detecting and measuring clinically significant biomarkers. Humanity is confronted with growing risks from emerging and recurring infectious diseases, including the influenza virus, dengue virus (DENV), human immunodeficiency virus (HIV), Ebola virus, tuberculosis, cholera, and, most notably, SARS coronavirus-2 (SARS-CoV-2; COVID-19), among others. Timely diagnosis of infections and effective disease control have always been of paramount importance. Plasmonic-based biosensing holds the potential to address the threat posed by infectious diseases by enabling prompt disease monitoring. In recent years, numerous plasmonic platforms have risen to the challenge of offering on-site strategies to complement traditional diagnostic methods like polymerase chain reaction (PCR) and enzyme-linked immunosorbent assays (ELISA). Disease detection can be accomplished through the utilization of diverse plasmonic phenomena, such as propagating surface plasmon resonance (SPR), localized SPR (LSPR), surface-enhanced Raman scattering (SERS), surface-enhanced fluorescence (SEF), surface-enhanced infrared absorption spectroscopy, and plasmonic fluorescence sensors. This review focuses on diagnostic methods employing plasmonic fluorescence sensors, highlighting their pivotal role in swift disease detection with remarkable sensitivity. It underscores the necessity for continued research to expand the scope and capabilities of plasmonic fluorescence sensors in the field of diagnostics.

## 1. Introduction

Infectious diseases, often termed as communicable diseases, result from harmful microorganisms like bacteria, viruses, fungi, or parasites, exhibiting the potential for rapid transmission and infection among human or animal carriers through methods such as inoculation, airborne dispersal, or waterborne spread [1]. They can spread either directly or indirectly from person to person, or even from animals or environmental factors to humans. Approximately 75% of emerging infectious diseases are zoonotic, meaning they can be transmitted between humans and animals, leading to about a billion cases of illness and millions of deaths per year [2]. While some of these diseases are benign and resolve on their own, others may cause intense medical conditions, lasting health issues, or even prove fatal, with significant global effects that impact not just individuals’ health but also that of societies, economies, and political systems. [3]. For centuries, these diseases have stood alongside wars and famine as formidable hurdles to the progress and survival of humanity [4]. From the devastating effects of the bubonic plague in the Middle Ages to the 1918 influenza pandemic, and more recently, the global outbreak of diseases like HIV/AIDS and COVID-19, the world has witnessed the profound consequences of these diseases [5]. The late twentieth and early twenty-first centuries have witnessed the swift spread and struggle to control numerous diseases like HIV/AIDS, malaria, and tuberculosis that continue to take millions of lives annually [6]. Others, including dengue fever and several types of hepatitis, are witnessing a rise in infections and fatalities. While diseases like measles and poliomyelitis are on the verge of elimination, the danger of recurrence is imminent if efforts to control them weaken. On the contrary, diseases like SARS, SARS-CoV-2, Ebola, H1N1 influenza, and Zika have either subsided or are under effective control [6]. Nonetheless, certain illnesses, like cholera, continue to pose recurrent hazards by causing frequent outbreaks. In managing infectious diseases, it is crucial to control the source of infection. This requires prompt detection, immediate isolation, and timely treatment of affected patients. Consequently, there is a pressing need for the development of fast, precise, and reliable detection methods and diagnostic tools [7,8]. Current traditional diagnostic methods, including the identification of microorganisms through culture observations, enzyme-linked immunosorbent assay (ELISA), reverse transcription polymerase chain reaction (RT-PCR), and quantitative real time-polymerase chain reaction (qPCR), are primarily conducted in medical laboratories [9]. These methods often have limitations, including being time-consuming, labor-intensive, costly, reliant on expensive infrastructure, and lacking the capability for swift on-site detection [1,10]. ELISA is one of the common immunoassay techniques designed for detecting and quantifying substances such as peptides, proteins, antibodies, and hormones. In ELISA, an antigen (the substance to be measured) is immobilized on a solid surface and then forms an immune complex with an antibody that is linked to an enzyme. Detection is accomplished by assessing the conjugated enzyme activity via incubation with a substrate to produce a measurable product. The most crucial feature of ELISA is that it provides specific measurements because the antigen-antibody reaction is highly specific. Until now, the optical ELISA has been highly successful and is the most widely used technique for screening target proteins in real samples [11]. Nevertheless, its limited sensitivity, the high cost of equipment, and lack of portability present significant challenges. The polymerase chain reaction (PCR) represents a lab technique designed for amplifying a designated DNA segment through an enzymatic reaction. Modifications to the fundamental PCR process have broadened its utility. The advent of qPCR permits the real-time detection and quantification of a specific sequence concurrently with its synthesis. Additionally, RT-PCR stands out as a method for detecting and quantifying RNA [12]. Among the available approaches for infectious disease diagnosis, molecular diagnostics demonstrate high sensitivity, while the industrial progress of microfluidics remains limited due to challenges like complex channel designs, costly materials, reaction optimization, leakage, and replication issues [10,13]. Surpassing the cumbersome and traditional in vitro methods used for diagnosing infectious diseases, biosensors have showcased remarkable potential in achieving ultra-sensitive detection of biomarkers associated with infectious diseases [14]. Various sensing approaches aimed at creating point-of-care (POC) devices that offer highly sensitive, swift outcomes and quantitative digital data via straightforward operations have been evaluated in recent years. This evaluation stems from the recognition that a POC diagnostic device, intended for decentralized testing, holds the promise of substantially reducing the time required for treatment, especially in the realm of infectious diseases [15]. In this regard, plasmonic-based biosensing presents an alternative approach that has received significant interest within the scientific community due to its remarkable sensitivity and promising potential as a novel method for swift, real-time and label-free detection of infectious diseases [16,17]. This technique also offers the benefits of user-friendly operation, minimal sample preparation requirements, and uncomplicated, cost-effective instrumentation. Additionally, fluorescence-based biosensors have found extensive application in life sciences and biomedical fields due to their low limit of detection and wide range of available fluorophores, enabling simultaneous detection and measurement of numerous biomarkers [18]. This fluorescence-based measurement method offers the advantage of signal enhancement using different plasmonic nanomaterials. By combining plasmonic nanomaterials with fluorescence molecules, a modification in fluorescence properties can be induced, resulting in intensified emission. The resulting enhancement of fluorescence due to proximity, recognized as metal-enhanced fluorescence (MEF), significantly amplifies the intensity of the fluorescent signal, offering the prospect of achieving highly sensitive detection of infectious diseases [19]. This review will thoroughly delve into techniques for fluorescence enhancement and showcase existing diagnostics that employ plasmonic-based fluorescence biosensing for the detection of infectious diseases.

## 2. Types of Infectious Disease

**Figure 1:**
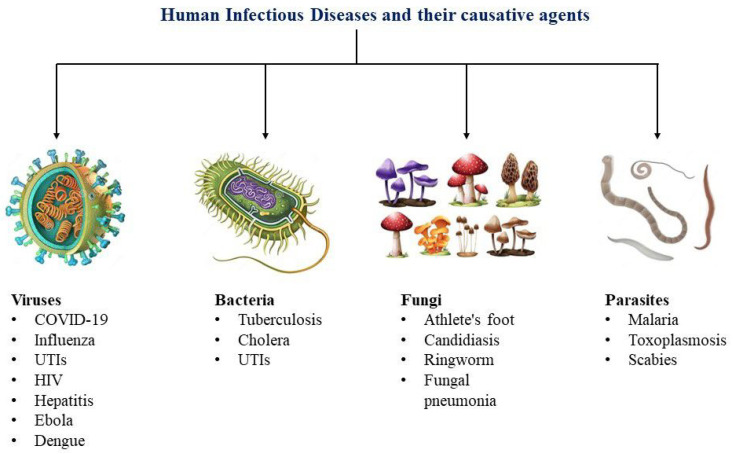
Human infectious diseases and their causative agents.

The recent emergence of COVID-19 outbreaks has put uncertainty on the existing global health system’s ability to provide adequate safeguards against an expanding and dynamically shifting spectrum of infectious disease risks. Furthermore, the occurrence of recent outbreaks such as Ebola, Zika, dengue, Middle East respiratory syndrome (MERS), severe acute respiratory syndrome (SARS), and influenza, alongside the imminent threat of escalating antimicrobial resistance (AMR) and a multitude of known and unidentified pathogens not only endangers human health but also poses significant risks to diverse aspects of societal and economic prosperity [20]. Infectious diseases remain a persistent threat to both humans and animals, leading to disability and fatalities as new disease-causing agents emerge and previously known pathogens reappear or undergo evolutionary changes. Viruses, including influenza, measles, and West Nile virus, as well as bacteria, such as anthrax, salmonella, chlamydia, cholera, and protozoa, encompassing malaria and trypanosomiasis (sleeping sickness), exemplify the diverse array of diseases that can be transmitted. Transmission routes include direct person-to-person contact through respiratory droplets (e.g., measles), transmission via bodily secretions (e.g., chlamydia), vector-borne transmission through biting tsetse flies (e.g., trypanosomiasis) or mosquitoes (e.g., malaria), or the ingestion of contaminated food or water (e.g., cholera) [21]. The expanding and constantly evolving range of infectious disease threats has underscored the urgent requirement for advancements in epidemiological testing devices that offer high-throughput capabilities, exceptional resolution, and superior clinical sensitivity and specificity [22]. While infectious diseases encompass a wide range of conditions, including fungal infections, parasitic infections, protozoal infections, and prion diseases, this review will primarily concentrate on viral and bacterial infections in humans. These two categories represent significant areas of study and public health concern, warranting a focused analysis to delve into the current understanding, advancements, and challenges surrounding viral and bacterial infectious diseases. Human infectious diseases and their respective causative agents are outlined in Figure 1.

### 2.1. Viral Infections

Viral infections have long been a significant global health concern, posing a considerable threat to both human and animal populations. These infectious agents, distinguished by their tiny genetic material contained within protein structures, demonstrate an exceptional ability to enter host cells and control their processes to replicate and spread. Viruses have pathogenicity levels equivalent to other microorganisms and are expected to evolve at a faster rate than protozoa, fungus, or bacteria. Relying on a multitude of host-encoded proteins, these obligate parasites are unable to find refuge in the extracellular environment, compelling them to continuously face immune responses across diverse host species [23]. Acute viral infection is characterized by continuous changes in both the host and virus until the infection is resolved, becomes chronic, or results in host mortality. Genes specific to acute infection play a crucial role, while chronic viral infection involves a dynamic equilibrium between viral and host genes. Understanding the distinct rules governing acute and chronic infection is essential, as chronic viruses persist despite the host’s immune defenses, presenting a challenge in immunobiology [24]. Upon viral infection, the immune system utilizes pattern-recognition receptors (PRRs), including Toll-like receptors (TLRs) and cytoplasmic RNA helicases, to detect viral components. TLRs recognize viral components both outside and inside cells, activating signaling pathways that lead to interferon production and inflammation. In contrast, cytoplasmic RNA helicases specifically recognize viral double-stranded RNA within cells, initiating their own signaling pathways for interferon production [25]. Efficient viral replication is dependent on enhanced cellular metabolism and adequate iron availability, emphasizing viruses’ reliance on iron-rich host cells for growth. Considering the viruses’ reliance on host cells for replication and their ability to manipulate iron homeostasis, as seen in HIV-1 and hepatitis C virus infections, studying the interaction between iron metabolism and viral infection could provide valuable insights and potentially lead to novel approaches in disease control [26].

#### 2.1.1. COVID-19

COVID-19, caused by SARS-CoV-2, is characterized by its efficient transmission and virulence. This viral infection has sparked a worldwide pandemic, leaving a significant impact on human populations. Genomic analysis has identified a phylogenetic connection between SARS-CoV-2 and bat viruses, particularly SARS-like strains, implying that bats may act as the primary reservoirs for the virus [27]. The global count of confirmed COVID-19 cases stands at 772,052,752, with 6,985,278 reported deaths to the World Health Organization in November, 2023 [28]. COVID-19 presents a diverse array of clinical manifestations, often encompassing flu-like symptoms such as fever, dry cough, shortness of breath, fatigue, headache, loss of smell, diarrhea, and frequently involving the respiratory system [29,30,31,32]. The interplay between the host’s immune response and SARS-CoV-2 holds a pivotal role in shaping disease trajectory and clinical expressions. In addition to triggering antiviral immune responses, SARS-CoV-2 can induce excessive inflammatory reactions, marked by a significant release of pro-inflammatory cytokines in severe COVID-19 cases. This might potentially lead to lymphopenia, lymphocyte dysfunction, alterations in granulocytes and monocytes, increased vulnerability to secondary infections, septic shock, and the onset of severe multi-organ dysfunction [33]. Furthermore, research indicates that severe COVID-19 is linked to elevated rates of autoantibodies against type-I interferons (IFNs), particularly prominent in male patients [34]. Coronaviruses are categorized into four main subgroups (α, β, γ, and δ), with the α group containing six members, including human pathogens Cov-229E and Cov-HKU1 [29]. SARS-CoV-2 is also classified within the β coronavirus category. The amino acid sequences in seven conserved domains of the genomic open reading frame 1ab (ORF1ab) show a significant 94.6% similarity to the original SARS-CoV. Furthermore, the SARS-CoV-2 virus, with a diameter of 60–140 nm, carries a single-stranded RNA genome of 29,891 base pairs, displaying a 79.5% sequence resemblance to SARS-CoV in genome alignment [35]. It is possible to classify the progression of SARS-CoV-2 into three primary stages: stage I entails an asymptomatic incubation phase, during which the presence of the virus may or may not be detectable; in stage II, there is a non-severe symptomatic period accompanied by the virus; lastly, stage III signifies the emergence of severe respiratory symptoms, accompanied by a substantial viral load [36]. The immune response to SARS-CoV-2 comprises two phases: an initial stage focused on controlling the virus and preventing severe disease, and a subsequent phase characterized by tissue damage and inflammation. Challenges in developing virus-clearing immunity after recovery highlight the importance of comprehensive vaccine strategies considering viral complexity and genetic variations [37,38]. In reaction to the seriousness of the symptoms, a variety of vaccines and medications have received emergency use authorization and are extensively administered to alleviate the impacts of COVID-19. Up to this point, several COVID-19 vaccines, including Pfizer-BioNTech, Moderna, Johnson & Johnson, AstraZeneca, Sinovac, Sinopharm and Bharat have gained authorization or approval for use. Alongside vaccines, various medications such as antiviral drugs, anti-inflammatory drugs, and treatments like hydroxychloroquine and ivermectin have been employed to manage COVID-19 symptoms [39]. The Pfizer COVID-19 vaccine (BNT162b2) received emergency use authorization from the WHO on 31 December 2020, followed by the AstraZeneca/Oxford vaccine, produced by the Serum Institute of India and SKBio, gaining approval on 15 February 2021. Recent additions include the Ad26.COV2.S vaccine, developed by Janssen (Johnson & Johnson) on 12 March 2021, and the Moderna vaccine on 30 April 2021 [40]. Following the initial vaccine dose, the collective effectiveness of all COVID-19 vaccines analyzed in the study in [41] was observed to be 71% (95% CI 0.65, 0.78). After the second dose, the overall effectiveness of the vaccines increased to 91% (95% CI 0.88, 0.94). The combined efficacy of vaccines following the first and second doses yielded values of 81% (95% CI 0.70, 0.91) and 71% (95% CI 0.62, 0.79), respectively. Among the vaccines examined, the Moderna vaccine exhibited the greatest effectiveness after both the initial and second doses, recording rates of 74% (95% CI, 0.65 to 0.83) and 93% (95% CI, 0.89, 0.97), respectively. Reference [41] offers a comprehensive overview of various vaccines and their efficacies, as well as the overall effectiveness of the alpha and gamma variants following the first and second dose among the vaccines studied [41].

#### 2.1.2. Influenza Virus

Every year, seasonal influenza viruses infect 5–15% of the global population, resulting in around 500,000 deaths worldwide [42]. There exist four categories of influenza viruses: A, B, C, and D [43]. The family Orthomyxoviridae includes five genera, with influenza A, B, and C viruses belonging to three of them. These viruses are distinguished by their genomes, which are segmented and consist of negative-strand RNA [24]. In terms of clinical impact, influenza A viruses are the most significant of the three varieties of influenza viruses (A, B, and C), as they are responsible for causing severe epidemics in both humans and domestic animals [44]. Influenza A viruses are further classified into subtypes based on the specific combination of haemagglutinin (HA) and neuraminidase (NA) glycoproteins found on their surfaces [42]. Currently, there exist 18 different subtypes of haemagglutinin (HA) and 11 subtypes of neuraminidase (NA) in influenza viruses. The majority of these subtypes are found circulating among wild birds. However, only three combinations, namely A/H1N1, A/H2N2, and A/H3N2, have been observed to widely circulate among humans. Among these combinations, A/H1N1 and A/H3N2 subtypes are the ones responsible for causing seasonal influenza virus epidemics [45]. While the existing influenza virus vaccines are effective in combating the disease, they provide limited and strain-specific immunity. These vaccines need to be regularly updated, which involves a complex, expensive, and time-consuming process, due to the continuous antigenic drift of the viruses [46]. Antigenic variants of A/H3N2 viruses emerge at intervals of approximately 3–5 years, while new antigenic variants of A/H1N1 and influenza B viruses appear less frequently, with intervals ranging from 2 to 5 years for A/H3N2 viruses compared to 3–8 years for A/H1N1 and influenza B viruses [47,48,49,50]. Upon infection with influenza viruses, the human body activates innate and adaptive immune responses [51], aiming to eliminate the infection. This involves the creation of strain-specific antibodies that exert selective pressure on the circulating viruses [52]. These responses also activate virus-specific T cells, including CD4+ T helper cells and CD8+ cytotoxic T cells [53]. CD4+ T cells contribute to B-cell responses [54], while CD8+ T cells, also known as cytotoxic T lymphocytes (CTLs), are recruited to infection sites to eliminate virus-infected cells, thereby preventing viral replication [51]. While animals infected with diverse strains of influenza viruses exhibited comparable findings [55], alternative studies conducted by Davenport and Hennessy [56] have suggested that prior exposures could potentially weaken the potency or efficacy of antibody responses towards new viral strains. While evidence of ‘antigenic sin’ in humans exists, there is still a growing need for further research to comprehensively grasp the mechanisms behind the initiation of human antibody responses and the impact of these antibodies on viral evolution and vaccine efficacy [57,58,59]. Various diagnostic methods are employed to detect influenza virus infections in humans, encompassing viral isolation in cell culture [60], immunofluorescence assays [61,62,63], serological assays [64,65], immunochromatography-based rapid diagnostic tests [66,67,68,69], nucleic acid amplification tests [70,71,72] and microchip devices [73,74,75,76,77], among other techniques. Multiple platforms are utilized in the production of influenza virus vaccines, encompassing whole-virus inactivated vaccines, split vaccines, subunit vaccines, live attenuated influenza vaccines (LAIVs), recombinant influenza virus vaccines, cell-culture-derived seasonal influenza virus vaccines, and virus-like particles (VLPs) [78,79,80,81,82]. Significant progress in influenza vaccine development since 2009 includes the adoption of innovative production techniques and enhanced formulations, all working towards the goal of producing lifelong, universally effective vaccines against all viral strains [83].

#### 2.1.3. Human Immunodeficiency Virus (HIV) Infection

Since the initial recognition of the disease in 1981, HIV/AIDS has caused over 700,000 fatalities in the United States [84]. According to the Centers for Disease Control and Prevention (CDC) estimates from 2019, there are approximately 1.1 million people currently residing in the country with HIV. Alarmingly, about 13% of these individuals are unaware that they are infected with the virus [85]. The current data suggest that the primary source of HIV transmission, accounting for over 90% of cases, stems from two groups: individuals diagnosed with HIV who are not actively engaged in medical care (69%), and individuals whose infection has not yet been diagnosed (23%) [86]. In 2017, the United States witnessed over 38,000 newly diagnosed cases of HIV. This alarming statistic revealed a significant concentration of cases among young men who have sex with men (MSM), as well as a notable incidence of HIV among transgender individuals, high-risk heterosexuals, and individuals who engage in drug injection practices [87]. Most HIV transmission cases arise from heterosexual contact with a partner who was unaware of or did not disclose their HIV status. Based on genetic characteristics and differences in the viral antigens, HIV is classified into the types 1 and 2 (HIV-1, HIV-2) [88]. HIV-1 originated from immunodeficiency viruses found in Central African chimpanzees, known as SIVcpz, while HIV-2 emerged from SIVsm, which is prevalent in West African sooty mangabeys [89]. HIV is a retrovirus that can integrate its DNA into the DNA of the host, making it highly resistant to current treatments. Once inside a cell, the virus converts its RNA into DNA, which becomes part of the host DNA. By utilizing the host’s enzymes, HIV undergoes transcription, protein production, cleavage, and releases mature virions [90]. HIV requires a co-receptor, generally CCR5 or CXCR4, to enter the host cell. One characteristic feature of HIV-1 infection is its significant rate of variation, with an estimated occurrence of approximately one mutation during each replication event. The combination of a high replication error rate with continuous, vigorous viral replication results in a great degree of variety within HIV-1. The immune system and antiretroviral medications exert selective pressures that impact the survival and spread of particular HIV-1 variants [91]. In response to HIV infections, the testing algorithms have developed over time as test accuracy has improved. Antiretroviral therapy (ART) has been available as a treatment for HIV infection for nearly two decades. It is highly recommended to initiate ART as early as possible following the diagnosis of HIV [92]. When used correctly, ART has shown extraordinary efficacy in fully or nearly completely blocking HIV replication, improving immunological function, and dramatically lowering the risk of developing AIDS. However, it is vital to highlight that ART does not provide an HIV cure and if discontinued, the virus inevitably rebounds within a matter of weeks [93].

#### 2.1.4. Hepatitis

Hepatitis is a term used to describe inflammation of the liver. Viral hepatitis (VH) has existed for as long as human civilization, spanning throughout recorded human history [94]. It can be caused by various factors, including viral infections, alcohol abuse, certain medications, toxins, and autoimmune diseases. However, viral hepatitis is the most common form and is primarily caused by five different viruses: hepatitis A (HAV), hepatitis B (HBV), hepatitis C (HCV), hepatitis D (HDV), and hepatitis E (HEV). In this comprehensive review, we will provide a concise overview of the three most prevalent variants of hepatitis, namely, HAV, HBV, and HCV. Hepatitis A is typically transmitted through contaminated food or water or close contact with an infected person. HAV infection is the primary cause of acute hepatitis and acute liver failure in children in numerous countries. HAV is a tiny, non-enveloped RNA virus that is 27 nm in diameter. It is a member of the Picornaviridae family and has a single serotype. The virus replicates in the liver, is expelled in bile, and can be found in feces [95]. Hepatitis A can manifest as symptomatic or asymptomatic, with older individuals more likely to exhibit symptoms, including jaundice. The disease follows three phases: incubation, symptomatic infection, and convalescence. Hepatitis A virus is excreted in feces during the symptomatic phase [96], and diagnosis relies on markers like anti-HAV IgM (detectable 5–10 days after exposure, up to 6 months) and anti-HAV IgG, which remains detectable for life, providing lifelong protection against reinfection. Acute hepatitis A lasts one to several weeks, with symptoms such as fever, malaise, loss of appetite, headache, and potential jaundice. Chronic hepatitis B (CHB) infection is the most prevalent viral infection worldwide [97], affecting nearly 300 million people globally, and causing nearly 1 million deaths annually due to complications like liver cirrhosis and hepatocellular carcinoma (HCC) [98]. The transmission of HBV occurs through contact with blood or semen that is infected with the virus. Perinatal transmission occurs from infected mothers to newborns in high-endemic areas, while sexual transmission is common in low-endemic areas, especially among individuals with multiple sexual partners, men who have sex with men (MSM), and those with a history of sexually transmitted infections. Unsafe injections, blood transfusions, or dialysis also pose a risk for HBV transmission [97]. During the early stages of HBV infection, PCR can detect the virus in the blood within about a month. Virus levels remain relatively low for up to six weeks before rising to their peak. At this time, specific substances called HBV e antigen (HBeAg) and HBV surface antigen (HBsAg) are also present in higher amounts. Additionally, specific antibodies against HBV core antigen (HBcAg) can be detected early on and remain in the body for life, regardless of the outcome of the infection. Around 10 to 15 weeks after infection, the levels of an enzyme called alanine aminotransferase (ALT) start to rise, indicating that the immune response is causing damage to the liver cells. Interestingly, most adults who are acutely infected with HBV recover fully from the infection, develop antibodies against HBeAg and HBsAg, and clear these antigens from their blood. It is important to note that when HBV infection occurs during infancy or childhood, it often leads to a chronic form of hepatitis, which is a long-term inflammation of the liver [99]. Unlike HBV, HCV rapidly increases in the bloodstream and reaches high levels within one week of infection. When compared to HBV, the immune response to HCV is delayed, with adaptive cellular responses taking at least one month and humoral responses taking at least two months. Symptoms such as jaundice, which are induced by T-cell-mediated liver damage in acute hepatitis B, are uncommon in HCV infection [100]. After the first week of HCV infection, the rate of viral increase slows down, and the peak viral levels are much lower than in acute HBV infection [101]. Around 8–12 weeks after infection, when ALT levels peak, HCV RNA levels start to decline. Only a small proportion of patients recover completely and test negative for HCV RNA. Viral clearance from the liver and other reservoirs may take longer than clearance from the blood, and antiviral therapy is considered for all patients with chronic HCV infection [102].

#### 2.1.5. Ebola Virus Disease

Ebola virus disease (EVD) is a severe and often deadly illness caused by the Ebola virus. The seven filoviruses identified in human populations are classified into two genera: Ebolavirus and Marburgvirus. The Ebolavirus genus includes Bundibugyo virus (BDBV), Ebola virus (EBOV), Reston virus (RESTV), Sudan virus (SUDV), and Taï Forest virus (TAFV). The Marburgvirus genus comprises Marburg virus (MARV) and Ravn virus (RAVV). While both genera cause severe hemorrhagic fevers in humans and non-human primates, Marburgviruses generally have a longer incubation period and a slightly different symptom profile compared to Ebolaviruses, reflecting variations in their viral structures and pathogenesis mechanisms [103]. EVD is a specific illness that is exclusively caused by the EBOV [104]. Between late 2013 and early 2016, EBOV triggered the most significant outbreak on record, originating in Guinea and subsequently spreading to other nations in Western Africa, resulting in a total of 28,652 reported cases of human infections and 11,325 fatalities [105]. Ebola viruses are primarily transmitted among humans through direct contact with various sources such as infected blood, secretions, tissues, organs, and other bodily fluids originating from symptomatic or deceased individuals infected with Ebola virus disease (EVD) [106,107]. The infection typically leads to tissue damage, particularly in the liver, spleen, kidney, lymph nodes, testes, and ovaries, as the virus replicates within the cells of these organs [108]. This replication process is associated with microvascular damage, alterations in vascular permeability, and activation of the clotting cascade [109]. Platelets and endothelial cells are also affected, disrupting fluid balance and overall homeostasis [108]. Furthermore, the virus is thought to compromise and suppress the immune system’s functionality [109]. In most cases, the sickness proceeds in three stages: an initial phase marked by nonspecific symptoms like fever, headache, and muscle soreness, followed by a gastrointestinal phase featuring symptoms such as diarrhea, vomiting, abdominal discomfort, and dehydration. The second week may bring improvement or a deterioration in the patient’s condition, leading to the third stage characterized by severe signs including collapse, neurological issues, and bleeding, often resulting in fatality [109]. EBOV targets specific cells like mononuclear phagocytes and dendritic cells, causing the release of pro-inflammatory cytokines and contributing to immune cell depletion and severe conditions like hypotension, disseminated intravascular coagulation, and multiple organ dysfunction syndrome in EVD patients [110]. A positive Ebola RT-PCR is the primary test used to confirm Ebola virus infection. The results of the RT-PCR test are available within 24–48 h, faster than the ELISA test [110]. The swift isolation of Ebola patients and proper use of protective gear by healthcare workers are crucial for outbreak control. In cases of exposure to body fluids from a potentially infected patient, thorough cleaning is essential, including disinfecting or disposing of contaminated personal belongings and patient’s home [111]. The most advanced vaccines in the United States and Europe are Ervebo (rVSV-EBOV), Zabdeno/Mvabea (Ad26-ZEBOV/MVA-BN-Filo), and cAd3-EBOZ. These vaccines primarily target the EBOV species but have not been specifically studied for their effectiveness against other Ebola virus species, such as SUDV, BDBV, or TAFV [112].

#### 2.1.6. Dengue Fever

Dengue virus (DENV) poses a substantial global health menace, with millions of infections occurring each year in tropical and subtropical areas worldwide especially in Asia and South America. The dengue virus (DENV), a member of the Flaviviridae family and Flavivirus genus, is, a positive (+) stranded RNA containing virus and primarily transmitted to humans through Aedes mosquitoes, particularly Aedes aegypti. The dengue virus exhibits antigenic diversity and is classified into four distinct serotypes, namely DENV1–DENV4. Additionally, a fifth serotype (DENV-5) was identified through isolation and genetic sequence analysis in Sarawak state of Malaysia in October 2013 [113]. The dengue virus poses a significant threat to human life, causing severe illnesses such as dengue hemorrhagic fever (DHF) and dengue shock syndrome (DSS), which are characterized by the abnormal leakage of plasma from blood vessels, leading to potential complications such as severe hemorrhaging, organ malfunction, and shock [114]. With approximately 400 million infections and 22,000 deaths occurring annually, its impact on global health is substantial [115]. Following an infection with a specific serotype, individuals develop immunity against re-infection by the same serotype. However, because the cross-immunity is only temporary, further infections with different serotypes are possible. Extensive cohort studies have revealed that protective immunity against heterotypic serotypes gradually declines over the course of one or two years [116]. Dengue fever (DF) is a febrile disease with flu-like symptoms, but asymptomatic infections are also common. According to transmission dynamics models, the majority of illnesses are asymptomatic [117]. It typically includes symptoms such as a sudden onset of high fever accompanied by an intense headache, pain behind the eyes, muscle and joint aches, gastrointestinal discomfort, and often a rash. In some cases, minor bleeding symptoms like small red spots (petechiae), nosebleeds (epistaxis), and bleeding gums (gingival bleeding) may occur. DF is typically associated with a decrease in the white blood cell count (leukopenia), while a decrease in platelet count (thrombocytopenia) may occasionally be observed, especially in individuals with hemorrhagic signs [118]. The immune response to dengue virus involves multiple cell types and mechanisms. Cells that are directly infected with the virus generate innate immune responses, including the production of type I and type II interferons. Cytotoxic lymphocytes play a role in eliminating infected cells, and B cells produce neutralizing antibodies that can counteract the virus [119]. In dengue virus infection, T cells play a crucial role by becoming active as T helper 1 (TH1) cells, contributing to the immune response [120]. Vaccination against dengue virus also triggers a TH1-cell response in the blood, as indicated by cytokines. Neutralizing antibodies, produced through collaboration between CD4+ T helper cells and B cells in the germinal center reaction, are effective in preventing dengue virus infection. The treatment for dengue fever primarily focuses on managing symptoms, such as using tepid sponging for fever and taking antipyretic medications for pain and fever relief. There is currently no specific antiviral drug available for dengue. However, studies have explored the potential antiviral activity of certain sulfated polysaccharides extracted from seaweeds against DENV. Additionally, nucleoside analogs like ribavirin and 6-azauridine have been investigated for their ability to reduce dengue virus activity in host cells by inhibiting nucleoside biosynthesis and protein synthesis, respectively. Other compounds such as curcumin, glycyrrhizin derivatives, and nucleoside adenosine analogs have also shown potential antiviral effects against dengue virus [115].

### 2.2. Bacterial Infections

Bacterial infections are a common type of infection caused by harmful bacteria that enter the body and multiply, leading to various illnesses and health complications. Bacteria are microscopic organisms that can be found in different environments, including the human body. While many bacteria are harmless or even beneficial, certain strains can cause infections when they invade tissues, organs, or systems. Bacterial infections are the primary cause of infectious diseases, although infections can also result from viruses, fungi, parasites, and protozoa [121]. Bacterial infections can be transmitted through five primary modes: contact, airborne, droplet, vectors, and vehicular [122]. While they can spread through direct and indirect contact with infected individuals or contaminated surfaces, they can also be spread through the air, either by inhaling aerosolized particles or respiratory droplets expelled during coughing, sneezing, or talking. Bacterial transmission can also occur through arthropod bites, such as mosquitoes or ticks, as well as through contaminated inanimate objects like food, water, or surfaces, which act as vehicles for bacterial spread.

#### 2.2.1. Tuberculosis

Tuberculosis (TB) is a global health concern, responsible for a significant number of fatalities with approximately 10.6 million reported cases and 1.6 million deaths in 2021 [123]. Advancements in our knowledge of tuberculosis TB have brought about a reevaluation of the conventional categorization of infected individuals into two distinct groups: those with latent tuberculosis infection (LTBI) and those with active TB disease. While active TB disease manifests with clinical symptoms and can affect multiple organs, latent tuberculosis infection is an asymptomatic state that is not transmissible. Although Mycobacterium tuberculosis (Mtb), the bacterium responsible for TB, can infect various parts of the body, pulmonary TB is the primary form that can be transmitted to others [124]. Mtb infection begins when a few bacilli are inhaled and reach the lungs. They are initially phagocytized by alveolar macrophages, but if they survive, they replicate and spread to nearby cells. If the bacilli survive, they replicate within the macrophages and spread to nearby cells, including epithelial and endothelial cells [125]. The bacterial burden increases, and Mtb can disseminate to other organs. The immune response forms granulomas which encapsulate Mtb bacteria. The granulomas act as a protective barrier, isolating the bacilli and limiting their interaction with the host immune system. Traditionally, LTBI was believed to occur when bacilli remained dormant inside granulomas, but recent studies show that Mtb can persist outside granulomas in different organs and tissues. During latent infection, most bacilli are dormant, but some actively replicate. If the immune response fails to control replication, active disease occurs. Factors like HIV, medications, and T-cell conditions disrupt the host–bacilli equilibrium, increasing TB risk [126]. Ensuring the timely diagnosis of TB is crucial for improving patient outcomes. However, a significant number of TB cases remain undiagnosed or unreported each year, posing a challenge to effective disease management and control efforts [127]. TB diagnosis involves detecting Mtb using microbiological techniques such as microscopic analysis, culture isolation, and molecular methods. These tests have high sensitivity and specificity, especially for pulmonary TB [127]. The detection of Mtb in urine or stools can be useful for diagnosing systemic infections, particularly in HIV-infected individuals and immunocompromised patients. Immunological diagnosis, such as the tuberculin skin test (TST) and interferon-gamma release assays (IGRAs), are also used to detect Mtb infection but cannot differentiate between LTBI and active TB. The Bacille Calmette–Guérin (BCG) vaccine is the oldest and most widely used vaccine for TB. It has been available for over a century and is primarily administered to infants and young children in high TB prevalence countries. The BCG vaccine provides some protection against severe forms of TB in children, but it does not offer complete protection against TB. Additional preventive measures and treatments are necessary to control the spread of the disease [128].

#### 2.2.2. Cholera

The bacterial infection known as cholera is caused by a microorganism called Vibrio cholerae, which is frequently present in contaminated food and water. This particular bacterium exhibits a curved and comma-shaped morphology, and it is classified as Gram-negative [129]. The described condition is an extremely contagious and rapidly spreading bacterial infection that arises from the colonization and proliferation of Vibrio cholerae within the intestinal tract. Individuals at high risk of infection acquire it by consuming a sufficient amount of the bacteria from contaminated water, vegetables, and food sources. Cholera is estimated to have an annual incidence ranging from 1.3 to 4.0 million cases, leading to a global mortality rate of 21,000 to 143,000 deaths [130]. Cholera outbreaks are often linked to various factors such as seasonal patterns, travel, natural disasters, armed conflicts, and specific circumstances that contribute to inadequate sanitation and widespread poverty [131]. A small proportion (10–20%) of individuals infected with cholera exhibit symptoms, typically presenting as mild acute watery diarrhea (AWD), which can be effectively managed with oral rehydration solutions. Approximately 20% of symptomatic cases, however, progress to a severe form characterized by sudden onset of copious watery diarrhea and vomiting, leading to dehydration. Without prompt and appropriate intravenous rehydration, correction of electrolyte imbalances, and administration of antibiotics, severe cholera cases can rapidly escalate and result in fatal outcomes [132]. Cholera vaccines are primarily classified into two categories: oral vaccines and injectable vaccines. Each type offers distinct characteristics and modes of administration to combat cholera. In comparison to injectable vaccines, killed oral cholera vaccines (OCVs) possess a significant advantage of being mucosal vaccines. These vaccines have the capability to stimulate immune responses specifically at the intestinal surface, which serves as the primary site of colonization by Vibrio cholerae. The first major oral cholera vaccine to be developed was Dukoral, which comprises inactivated Vibrio cholerae bacteria along with a component of cholera toxin. Additionally, Shanchol and Euvichol are also cholera vaccines that contain inactivated Vibrio cholerae bacteria but lack the cholera toxin component [130]. Killed OCVs have shown efficacy in the field, but they have considerable drawbacks. These vaccines routinely show low immunogenicity and limited protective efficacy in children under the age of five, who are at the greatest risk of serious cholera complications and fatality. Furthermore, there is a typical delay of one to two weeks between vaccination and the start of immunity. Injectable live attenuated (OCVs), currently represented by Vaxchora as the only available option, mimic natural infection with Vibrio cholerae and have the potential to address various limitations associated with killed OCVs. However, Vaxchora has not obtained WHO prequalification, partly due to its inadequate performance in a large-scale field efficacy study conducted in Indonesia [133].

#### 2.2.3. Urinary Tract Infections

Urinary tract infections (UTIs) are prevalent bacterial infections that impact millions of individuals worldwide. In 2019, global UTI cases reached 404.61 million, resulting in 236,790 deaths and 520,200 DALYs (disability-adjusted life years). Over the span of 1990 to 2019, there was a 2.4 fold increase in deaths, accompanied by a rise in the age-standardized mortality rate (ASMR) from 2.77 per 100,000 to 3.13 per 100,000 [134]. UTIs pose a substantial burden of morbidity in infants, older men, and females across all age groups [135]. Notably, UTIs are twice as likely to occur in females compared to males, and their prevalence escalates with advancing age [136]. In fact, it is estimated that approximately one in three women will experience at least one episode of UTI during their lifetime [137]. UTIs are caused by various bacteria, including Gram-negative, Gram-positive, and certain fungi. The primary causative agent for both uncomplicated and complicated UTIs is uropathogenic *Escherichia coli* (UPEC). In uncomplicated UTIs, UPEC is followed in prevalence by *Klebsiella pneumoniae*, *Staphylococcus saprophyticus*, *Enterococcus faecalis*, group B Streptococcus (GBS), *Proteus mirabilis*, *Pseudomonas aeruginosa*, *Staphylococcus aureus*, and *Candida* spp. On the other hand, for complicated UTIs, following UPEC as the most common agent, *Enterococcus* spp., *K. pneumoniae*, *Candida* spp., *S. aureus*, *P. mirabilis*, *P. aeruginosa*, and GBS are the subsequent prevalent causative agents [135]. A three-day regimen of trimethoprim-sulfamethoxazole, which provides a 90% eradication rate, is the gold standard for treating uncomplicated acute cystitis [138]. Ciprofloxacin, levofloxacin, norfloxacin, and gatifloxacin also provide comparable eradication rates after three days of treatment [139]. Fosfomycin tromethamine can be given as a single dose, while nitrofurantoin monohydrate macrocrystals require a seven-day, twice-daily regimen. Preventive measures for UTIs encompass maintaining proper hygiene practices, including wiping from front to back after toilet use [136], urinating before and after sexual activity, and ensuring adequate hydration. These strategies aim to minimize the risk of bacterial contamination and subsequent infection in the urinary tract.

### 2.3. Additional Infectious Pathogens

This section delves into a diverse group of infectious agents beyond viral and bacterial infections. It focuses on the intriguing domain encompassing fungal, parasitic, and prion pathogens. These infectious agents present unique challenges in terms of their transmission, clinical manifestations, and management. Understanding the characteristics, epidemiology, and impact of these diverse pathogens is crucial for comprehensive knowledge of infectious diseases. Fungal diseases, caused by a variety of fungal species, pose significant challenges to human health. These infections can affect different parts of the body, including the skin, nails, respiratory system, and internal organs. Understanding the nature of fungal diseases, their modes of transmission, and the available diagnostic and treatment strategies is crucial for effective management and prevention of these often persistent and recurrent infections. Some examples of fungal infections include athlete’s foot [140,141,142], candidiasis (yeast infection) [143,144], ringworm [145], and fungal pneumonia [146]. Parasitic infections, resulting from various parasites, have a substantial impact on global health, particularly in regions with limited access to adequate sanitation and healthcare. These infections can manifest in diverse forms, affecting different organs and systems within the human body. Parasitic infections encompass a variety of conditions, such as malaria [147,148], toxoplasmosis [149,150], and Scabies [151,152,153]. Prion diseases represent a distinct category of neurodegenerative disorders characterized by the abnormal transformation of normal cellular proteins into an infectious form. These conditions can result in profound neurological impairments and often have a fatal outcome. Unraveling the mysterious nature of prion diseases, comprehending their modes of transmission, and investigating potential therapeutic interventions remain active areas of research within the realms of neurology and infectious diseases. Notable prion diseases include Creutzfeldt–Jakob disease (CJD) [154,155], variant Creutzfeldt–Jakob disease (vCJD) [156], and Gerstmann–Sträussler–Scheinker syndrome (GSS) [157].

## 3. Methods for Fluorescence Enhancement

Fluorescence is a widely used phenomenon in various scientific and technological fields, including biology, chemistry, medicine, and materials science. The fluorescence signal provides valuable information about molecular interactions, concentrations, and structural changes. Fluorescence exhibits numerous valuable applications, encompassing the detection of single molecules [158], fluorescence nanoscopy [159], biological labeling [160], and optoelectronic device functionality [161], among many others. Fluorescence enhancement is imperative for enhancing the sensitivity and precision of a wide range of scientific and technological applications that heavily rely on fluorescence detection. It is a crucial requirement to elevate the performance and reliability of these applications. This review mainly focuses on technologies for plasmon-based fluorescence enhancement, even though other significant technologies such as photonic crystal-based fluorescent enhancement are included.

Fluorescence sensors operate on the principle of fluorescence, a photophysical process where a molecule, known as a fluorophore, absorbs light at a specific wavelength and then emits light at a longer wavelength. The mechanism involves the excitation of electrons to a higher energy state followed by their return to the ground state, accompanied by the emission of light. This characteristic emission is highly sensitive to the local environment of the fluorophore, making fluorescence sensors powerful tools for detecting specific ions, molecules, or changes in the physical conditions of their surroundings [162]. Enhancement and quenching effects are two critical phenomena in the operation of fluorescence sensors [163]. Enhancement refers to the increase in fluorescence intensity, which can be due to various factors, including changes in the fluorophore’s environment that stabilize the excited state or reduce non-radiative decay pathways, thereby increasing the efficiency of fluorescence emission [164]. Molecular interactions that lead to a more rigid fluorophore environment can also enhance fluorescence by restricting internal rotational movements that would otherwise dissipate energy non-radiatively [165]. Quenching, on the other hand, involves the decrease in fluorescence intensity [166]. Quenching mechanisms can be dynamic, where the quencher molecule interacts with the fluorophore in its excited state, or static, where the fluorophore and quencher form a non-fluorescent complex [167]. Quenching is particularly useful in fluorescence sensors for detecting the presence of quencher molecules, as the reduction in fluorescence directly correlates to the concentration of the quencher. Both enhancement and quenching effects are exploited in the design of fluorescence sensors to achieve high sensitivity and selectivity towards specific analytes. By understanding and manipulating these effects, advanced sensors can be developed for a wide range of applications, from medical diagnostics to environmental monitoring.

### 3.1. Fundamentals of Surface Plasmons

The study of plasmonics focuses on the collective electron oscillations in metallic media, known as “plasmons”, and their interactions with external electromagnetic fields, referred to as “polaritons”. Within this domain, plasmon polaritons are categorized primarily into two types: surface plasmon polaritons (SPPs) and localized surface plasmons (LSPs) [168]. SPPs are electromagnetic fields that propagate along the interface between metal and dielectric materials, while LSPs represent oscillations of electrons confined to the surface of isolated metallic nanostructures [169]. Metallic nanostructures demonstrate exceptional optical characteristics, primarily due to the excitation of their surface plasmons when exposed to light. This interaction significantly enhances the electromagnetic field at the nanoparticles’ surfaces. This enhanced near-field effect is useful in building very sensitive chemical and biosensors that can be finely controlled by manipulating the shape of the nanoparticles. Metallic nanoparticles have been found to increase fluorescence emissions while decreasing the excited-state lifetimes of adjacent fluorophores. The observed rise in fluorescence is due to a number of causes, including increased absorption by the fluorophore, changes in the molecule’s radiative decay rate, and better efficiency in coupling the fluorescent emission into the far field [170]. Generally, metallic nanoparticles that exhibit LSP resonances are preferred over metallic layers or surfaces that support propagating modes, known as SPPs. This preference stems from LSPs’ simpler coupling with excitation and emission light beams, as well as their relative lack of sensitivity to geometry or momentum conservation restrictions. This versatility allows LSPs to be used in a variety of configurations [171]. In contrast, while uniform layers that enable SPP modes have been used, there has been less research on connected metallic surfaces such as hole gratings. Importantly, the opto-plasmonic features of such structures arise from the interaction of LSP and SPP modes, resulting in the development of mixed modes such as Fano resonances [172]. Upon examining the transmission behavior relative to the characteristics of the electric field, it has been observed that the spectral region with the most significant relative enhancement aligns closely with the most pronounced indicators of plasmonic modes. Moreover, the mechanism of field coupling displays a notable directional quality in the propagation of the electric field, moving from the substrate’s backside to the nanostructured surface on the top and in the reverse direction. This directional behavior facilitates the effective design of the structure for optimal pumping and collection, making it highly suitable for sensing applications. This characteristic becomes crucial in the context of utilizing a plasmonic structure for the creation of a sensing device that leverages surface-enhanced fluorescence.

### 3.2. Plasmon-Enhanced Fluorescence (PEF)

#### 3.2.1. Metal-Enhanced Fluorescence (MEF)

**Figure 2:**
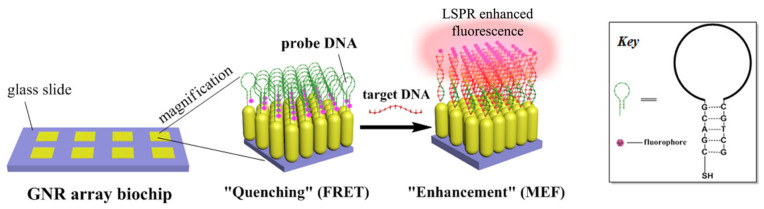
Schematic of the ordered GNR array chip for DNA detection via surface plasmon-enhanced fluorescence upon hybridization. Reprinted (adapted) from [176], Copyright (2017), with permission from American Chemical Society.

Plasmon-enhanced fluorescence (PEF) technology utilizes the increased electromagnetic (EM) field intensity near metal nanostructures to significantly enhance molecular fluorescence [173]. Metal-enhanced fluorescence (MEF) can be explained by two key factors. Firstly, it is attributed to the enhancement of excitation through the presence of strong local electric fields resulting from the excitation of localized surface plasmon resonance (LSPR). Secondly, MEF involves an increase in the radiative decay rate originating from the excited state of the fluorophore, which is coupled with surface plasmon resonance [174]. Despite its ease of implementation, LSPR biosensing frequently demonstrates constrained spectral sensitivity, presenting a hurdle for practical LSPR sensors that solely depend on spectral shifts for detecting biomolecules at ultra-low concentrations [175]. Tang et al. demonstrated that Fe_3_O_4_ magnetic nanoparticles (MNP) can significantly boost LSPR of metal nanoparticles. This enhancement is attributed to the high refractive index and substantial molecular weight of the Fe_3_O_4_ MNPs, rendering them potent enhancers for the plasmonic response to biological binding events. Using Fe_3_O_4_ MNPs to enhance LSPR assays, their practicality is tested with cardiac troponin I (cTnI) as a model protein for diagnosing myocardial infarction. This resulted in six-times-stronger spectral responses compared to direct cTnI adsorption on the GNR sensor, lowering the detection limit to approximately 30 pM in plasma samples. As a result, this breakthrough facilitates a notable enhancement in sensitivity, reliability, dynamic range, and calibration linearity when conducting LSPR assays to detect minute quantities of small molecules. However, it is important to note that fluorescence quenching effects have limited the progress of PEF in sensitive applications [164]. To address this, an innovative gold nanorod (GNR) array biochip as shown in Figure 2 was developed to systematically explore the enhancement of LSPR-coupled fluorescence [176]. The ordered assembly of GNRs on a glass surface dramatically intensified LSPR between adjacent nanoparticles. This resulted in a surface-plasmon-enhanced excitation and radiative mechanism for signal amplification. The appropriate choice of GNR size allowed for tunability, enabling overlap with the fluorophore’s excitation and emission wavelengths greater than 600 nm. The fluorescence enhancement was found to be distance-dependent, with the GNR array effectively overcoming quenching even at close proximity by fine-tuning the distance between the fluorophore and nanoarray surface. The enhancement correlated with the spectral overlap between the fluorophore and the plasmonic resonance of the GNR array, which is crucial for optimizing the efficiency of the process [177]. Through the utilization of the GNR array chip, fluorescence enhancement has led to the establishment of a detection limit at 10 pM, a value notably below that achieved by conventional LSPR aptasensors reliant solely on spectral shifts. These findings demonstrate the potential of the GNR array chip for practical and highly sensitive plasmonic DNA biochip applications. A further implementation of MEF was observed, specifically in the form of enhanced surface plasmon-coupled emission (SPCE), achieved by employing a hollow plasmonic structure [178]. Due to its distinctive features, including signal enhancement, distance dependence, and background suppression, SPCE holds great promise in the fields of biosensing and bioimaging [179,180]. The process involves the assembly of gold nanoshells (GNSs) onto a gold substrate via electrostatic adsorption, followed by the application of a thin layer of fluorophores (approximately 30 nm) using spin-coating. The resulting SPCE fluorescence signals exhibited enhancements of 30- and 110-fold compared to normal SPCE and free space emission, respectively. The observed enhancements were a result of several factors, including the formation of a nanostructure platform with a uniformly distributed region known as a plasmonic hotspots (where the electromagnetic field is significantly amplified) between GNSs and the gold substrate, the generation of an intense electromagnetic field by the GNSs, and the strong interactions between fluorescence and surface plasmons. This approach using hollow nanoparticles provides a convenient means to enhance the fluorescence signal, thereby improving the detection sensitivity in fluorescence-based sensing and imaging platforms [181].

**Figure 3:**
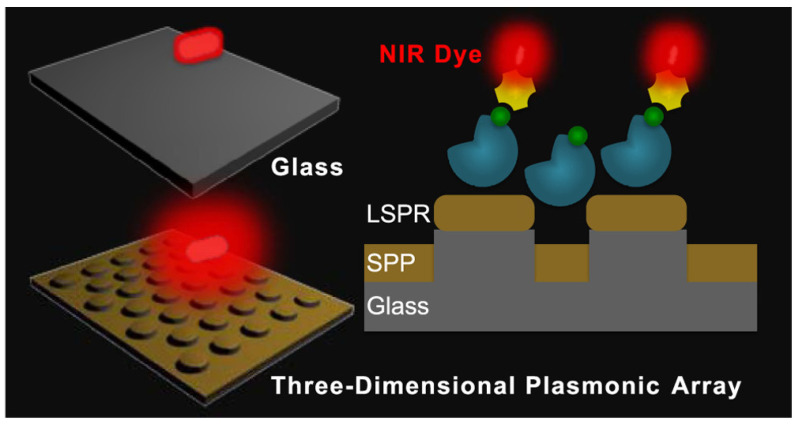
Three-dimensional (3D) gold nanohole-disc arrays (Au-NHDAs). Reprinted from ref. [182], Copyright (2019), American Chemical Society.

The development of multifunctional and multiplexed MEF platforms in the near-infrared (NIR) range holds great value, as it offers the advantages of reduced autofluorescence and minimized photoinduced damage. The utilization of a cost-effective, nanosphere lithography-based technique for fabricating three-dimensional (3D) gold nanohole-disc arrays (Au-NHDAs), as shown in Figure 3, serves as another notable illustration of MEF [182]. These arrays consist of glass pillars on top of nanoholes in a thin gold film. The pillars’ top surfaces are covered with gold nanodiscs, and small gold nanoparticles (nanodots) are positioned on the pillar sidewalls. This produces uniform and reproducible Au-NHDAs with controlled structures and adjustable optical properties in the near-infrared (NIR) range. These Au-NHDAs exhibit significant NIR fluorescence enhancement (over 400 times) due to the 3D plasmonic structure, enabling strong coupling of surface plasmons through glass nanogaps. The enhancement factor varies with the nanodisc diameter while maintaining the same resonance peak and separation distance. The hotspots in [183] are created by pairs of collapsible nanofingers, allowing for adjustable gap sizes with high precision at the sub-nanometer scale. Through experimental investigations, optimal gap sizes of the hotspots are identified for different dielectric spacer materials to maximize plasmon-enhanced fluorescence [184]. Intensely amplified electromagnetic fields in hotspots can be achieved in the vicinity of plasmonic nanostructures, comprising metallic nanoparticles positioned in close proximity with sub-nanometer interparticle gaps.

MEF can improve nanoantennas by creating accessible gaps, enhancing nearby fluorophores’ fluorescence. The nanofabrication technique described in [185] combines electron beam lithography, planarization, etch back, and template stripping to produce large arrays of in-plane nanoantennas. This technique results in remarkable fluorescence enhancement (up to $10^{4}$ to $10^{5}$ times) and enables detection volumes at the nanoscale in 20 zL range. The study in [186] showed that the adhesion layer between the gold film and glass substrate strongly influences the fluorescence enhancement of single molecules. They achieved a record-high enhancement factor of 25 by using a titanium dioxide (TiO_2_) adhesion layer. Metallic nanoparticles can significantly impact the emission of nearby fluorescent molecules and materials. In the case of indocyanine green (ICG) dye molecules, the fluorescence enhancement can be remarkable when they are in close proximity to nanoparticles with matching plasmon resonance frequencies and large scattering cross sections [173]. To achieve optimal fluorescence enhancement, it is crucial to align the plasmon resonance frequency of the nanoparticle with the emission frequency of the molecule. By increasing the particle’s scattering efficiency and tuning the plasmon resonance, the fluorescence of the molecule can be enhanced by more than 50 times. Dragan et al. have compared simulation study with empirical study for MEF sensors [187]. The simulations using finite difference time domain (FDTD) techniques reveal a significant enhancement of the excitation field attributed to resonant plasmonic modes supported by the nanoparticle aggregates. In the experimental investigations using Rhodamine 6G dispersed in polymethylmethacrylate, a remarkable 423 fold increase in fluorescence is observed. These findings highlight the potential of nanoparticle aggregates as a cost-effective and scalable platform for the development of mass-produced fluorescent biosensors that harness MEF. The 2-color DNA assay combines MEF with microwave-accelerated DNA hybridization to analyze DNA fragment sequences in solution. Using the “Catch and Signal” technology, it enables the simultaneous recognition of two target DNA sequences in one well [187]. Fluorescent labels (Alexa 488 and Alexa 594) attached to single-stranded DNA (ssDNA) fragments act as biosensor probes, enhancing MEF. It is shown that microwave irradiation for 30 s greatly speeds up selective DNA hybridization at room temperature, increasing the rate by about 1000 fold. This DNA assay platform greatly improves quantitative analysis of genome DNA sequences, offering a fast and simplified biomedical platform for nucleic acid analysis. Surface modification utilizing core/shell configurations using either inorganic or organic ligands has been demonstrated as a highly effective approach for attaining the external enhancement of luminescence [188]. A crystal lattice match is crucial for enhancing luminescence in inorganic materials [189]. Using noble metals and Ln^3+^ doped materials in the core/shell configuration, which can be termed as an external approach, improves the emission. On the other hand, an internal approach involves modifying the crystal structure and introducing sensitizers, which involve inner adjustments to alter the local structure, local symmetry, and interaction with active ions to enhance luminescence.

The new nanoplasmonic structure, termed as disk-coupled dots-on-pillar antenna array (D2PA), and an optimized spacer achieved substantial fluorescence enhancements of 2970 fold on average and $4.5\times 10^{6}$ fold at hotspots [190]. It demonstrated excellent uniformity over a large sample area, various dye concentrations, and laser powers. The structure was cost-effectively fabricated on 400 wafers using nanoimprint, self-alignment, and self-assembly methods. The presence and interaction of metal components, including metal ions or metallic nanostructures, have a significant influence on the aggregation of nanocrystals (NCs) or fluorophores, leading to a phenomenon known as Aggregation-Induced Emission (AIE) [191]. In AIE, the aggregation process induced by these metal components causes a notable enhancement in the emission of fluorescence. The metal components can modify the radiative and non-radiative decay rates of the nearby fluorophores, resulting in increased fluorescence intensity, improved quantum yield, and other properties. Solvent-induced aggregation [192], assembly-induced enhancement [193], and ion-induced aggregation [194,195] can be categorized as enhancement based on properties, where the presence and interactions of metal components induce the aggregation of nanocrystals or fluorophores, leading to enhanced fluorescence signals.

#### 3.2.2. Plasmonic Photonic Crystal-Induced Fluorescence Enhancement (PPCIFE)

Research indicates that combining the effects of plasmonics and photonic crystals (PCs) can lead to fluorescence enhancement. For instance, a technique to enhance the luminescence of CsPbCl_3_ nanocrystals (NCs) by combining them with Ag plasmon and photonic crystals has been investigated [196]. Theoretical simulations and experimental analysis revealed that when the plasmon peak of the Ag film and the photonic stop band of polymethyl methacrylate opal photonic crystals (OPCs) were well matched with the emission peak of the blue CsPbCl_3_ NCs, notable fluorescence enhancements of approximately 50 fold and 20 fold were achieved, attributed to improved excitation and emission fields. Subsequently, by synergistically utilizing surface plasmon and photonic crystal effects, the luminescent intensity of CsPbCl_3_ nanocrystals in CsPbCl_3_/Ag/OPCs hybrids is significantly enhanced by over 150 fold, resulting in an estimated emission efficiency of 51.5%. The fluorescence enhancement can also be achieved by combining the effects of photonic crystals and gold nanoparticles (AuNPs) with plasmonic properties. This method relies on the use of gold nanoparticles on silica photonic crystal microspheres (SPCMs) to enhance the natural fluorescence of proteins without labeling [197]. Aflatoxin B1 (AFB1) was detected as a model molecule. AFB1-bovine serum albumin and anti-AFB1 monoclonal antibodies were immobilized on SPCMs and AuNPs, respectively. AuNPs greatly enhanced the fluorescence of the antigens on SPCM using near UV excitation. Electric field simulation showed a maximum near-field enhancement of 20, resulting from the combined effects of photonic crystal and AuNP plasmon. Extensive research has investigated the strategic combination of metal and dielectric plasmonics with the interface of photonic crystals called photonic crystal-coupled emission, leading to valuable biophysicochemical insights. It has been observed that the coupling of photonic crystals with Ag soret colloids, nanovoids, and Bloch surface waves enhances luminescence [198]. Additionally, further research on a synergistic approach that involves the use of plasmonic-silver nanoassembly and high refractive index Nd_2_O_3_ sources, commonly referred to as `Huygen’ sources, demonstrated effective light scattering. Fluorescence enhancements exceeding 1200 fold, coupled with directional and polarized emission, were utilized to detect Rhodamine B dye at attomolar levels within integrated cavity hotspots that experienced amplified electromagnetic field intensity. Arranging finite-sized hexagonal arrays of nanoapertures in a lattice pattern within a gold film enables highly directional and enhanced emission from single fluorescent molecules placed in the central aperture [199]. This arrangement leads to a remarkable increase in brightness, with enhancements of up to 40 times per molecule in the forward direction. Another approach demonstrates that a remarkable 52-fold increase in signal intensity is achieved by combining plasmonic fluor nanoparticles and photonic crystals with a fluorescent dye [200]. The interaction between nanoparticles and light, along with improved light collection and increased emission rate, contribute to this enhancement. The method is showcased by successfully detecting a specific protein using a sandwich immunoassay. The limit of detection achieved is 10 fg mL^−1^ in buffer and 100 fg mL^−1^ in human plasma, demonstrating a sensitivity nearly three orders of magnitude higher than that of standard immunoassays. Hybrid structures of silver and hydrophobic 3D photonic crystals were created to investigate fluorescence enhancements [201]. The interaction between localized surface plasmon resonance and the 3D photonic stop band resulted in highly tunable properties. The study focused on fluorescence enhancements of conjugated polymer and quantum dot materials based on the hybrid structures, achieving a maximum enhancement of 87 times compared to glass substrates.

### 3.3. Photonic Crystal-Based Fluorescence Enhancement

Photonic crystals (PCs) are materials with a periodic variation in refractive index that selectively control the propagation of light, thereby significantly altering the emission properties of embedded optically active materials [202]. PCs can be categorized into one-dimensional, two-dimensional, and three-dimensional structures based on their spatial variations in refractive indexes [203]. When one-dimensional photonic crystals (1DPCs) combined with nanoscaled ZnO particles were fabricated using the spin-coating technique, a noticeable enhancement in the fluorescence of the organic dyes, compared to films of 1DPCs or ZnO alone, was observed [204]. Additionally, modifying the nanoscaled ZnO particles with Poly (sodium 4-styrene sulfonate) (PSS) and Poly (allylamine hydrochloride) (PAH) through self-assembly revealed that the degree of fluorescence enhancement was influenced by the thickness of PSS and PAH. Colloidal photonic crystals (CPCs) significantly enhance fluorescence efficiency by optimizing structure and dye arrangement [205,206]. An E-F-E double heterostructure (E denotes the monolithic CPC with a periodicity overlapping the excitation wavelength, while F represents the monolithic CPC with a periodicity overlapping the emission wavelength), made from multilayer CPCs, achieved a thousand-fold fluorescence enhancement. This heterostructure trapped both excitation and fluorescence by coupling CPCs with multiple-beam interference through overlapping periodicity for excitation and matching periodicity for fluorescence [207,208]. Fluorescence detection can also be enhanced using magnetically responsive CoFe_2_O_4_@SiO_2_@Ag CPCs [209]. The fluorescence spectra of different fluorescent molecules, such as Rhodamine B (RB) and fluorescein isothiocyanate (FITC), were selectively enhanced by adjusting the magnetic field to tune the photonic band gap of the substrates. The composites achieved high enhancement factors of 12.6 and 17.6 fold for RB and FITC, respectively, when the photonic band gap matched the fluorescence emission wavelength of fluorescent molecules. A genetic algorithm was used to design two photonic crystals using Al_2_O_3_ and TiO_2_, with one crystal containing SiO_2_. The sandwiched crystal formed a Fabry–Perot cavity, achieving 14-fold excitation enhancement [210]. By controlling electric field radiation using photonic forbidden bands, fluorescence in a 3.18 µm layer was enhanced by 60 fold. Regional differences in enhancement were due to phase changes from varying optical path lengths. A specialized two-dimensional photonic crystal array structure is used to significantly enhance the fluorescence intensity of sulfur dioxide, achieving a sensitivity of 1.224 L·mg^−1^ in the UV band [211]. Experimental measurements of TiO_2_ used in conjunction with the photonic crystal structure demonstrate a 10-fold increase in intensity compared to samples without the photonic crystal structure at room temperature. To improve the sensitivity of the microfluidic sensor for rapid detection of Cu^2+^ content in lubricating oil, a fluorescence enhancement method is employed by incorporating SiO_2_ inverse opal photonic crystals (IOPCs) into the sensor [212].

### 3.4. Hydrogel-Based Fluorescence Enhancement

Hydrogels are soft materials characterized by their porous structure and ability to retain a high amount of water while maintaining a distinct three-dimensional structure when swollen [213]. Due to these properties, they share similarities with biological tissues, making them suitable for various applications in the fields of biomedical and bioinspired materials [214]. Luminescent Carbon Quantum Dot hydrogels (CQDGs) can be used for direct determination of silver ions (Ag^+^) [215]. Different types of Carbon Quantum Dots (CQDs) were employed, each with unique surface properties (*passive*-CQDs with carboxylic groups, *thiol*-CDQs, and *amine*-CDQs), to create hybrid gels with a low molecular weight hydrogelator (LMWG). The use of gels significantly enhances fluorescence and shows selectivity for Ag^+^ ions due to their interaction with carboxylic groups on the CQDs. Among them, the CQDGs with carboxylic groups on their surface showed the highest selectivity for detecting Ag^+^ ions, possibly because of Ag^+^ ions’ flexible coordination. The sensing platform relies on a strong Ag–O interaction, which leads to the quenching of photoluminescence of *pasivate*-CDQs (p-CQDs) through charge transfer. The method demonstrates a low detection limit (LOD) and quantification (LOQ) of 0.55 and 1.83 μg mL^−1^, respectively, and it has been effectively employed for the analysis of river water samples. A pH and mechano-responsive coordination polymeric gel is formed by reacting Mg^2+^ with N-(7-hydroxyl-4-methyl-8-coumarinyl)-alanine [216]. The gelation process results in 3D nanostructures that trap water, leading to the formation of a freeze-dried hydrogel with uniform ribbon-shaped fibers. The hydrogel displays a distinct UV-vis absorption transition and, notably, exhibits a substantial enhancement in fluorescence intensity along with a longer lifetime compared to the original ligand. The incorporation of modified carbon nanodots into a unique hydrogel derived from a low molecular weight salt enhances the gelation properties, significantly increases fluorescence, and enables the hybrid gel to exhibit promising sensitivity towards heavy metal ions, particularly Pb^2+^, making it suitable for sensing applications [217]. Hydrogel microbeads can act as a platform for a bacterial sensor to detect nitro compounds [218]. Green fluorescent protein-producing *Escherichia coli*, which were engineered to be sensitive to nitro compounds, were encapsulated within hydrogel beads based on poly(2-hydroxyethyl methacrylate) [poly(HEMA)]. The hydrogel acted to concentrate and enhance the fluorescent signals emitted by the bacteria. By incorporating 80 wt% MAETC into the hydrogel beads, the fluorescence intensity of the bacterial sensors significantly increased compared to beads without MAETC. Supramolecular hydrogel serves as a unique matrix to immobilize proteins, peptides, substrates, chemosensors, and mesoporous silica particles on solid supports [219]. The gel’s semi-wet conditions and 3D nanofiber network structure effectively trap these substances while maintaining their functionality, resulting in a remarkable increase in fluorescence intensity. This enhanced fluorescence provides valuable insights into molecular recognition and enzyme activity, enabling effective monitoring and study of biological events. A boronic acid-based anthracene fluorescent probe was functionalized with an acrylamide unit and incorporated into a hydrogel system for monosaccharide detection [220]. The hydrogel exhibited a significant enhancement in fluorescence intensity upon exposure to fructose, resulting in a 10-fold increase in fluorescence intensity for the detection of monosaccharides. By forming a protective “shell” on the surface of functionalized carbon nanoparticles, glucose has been shown to significantly enhance their fluorescence while limiting movement and reducing fluorescence loss [221]. This leads to a remarkable 70-fold increase in fluorescence intensity under optimal conditions and enables the detection of glucose in serum samples, demonstrating a low limit of detection (10 μM) and a linear response within the concentration range of 50 μM to 2000 μM. Protein-induced fluorescence enhancement is a powerful method that significantly enhances fluorescence intensity when a protein binds closely, allowing for high-resolution studies of molecular interactions without the need for protein labeling [222,223,224].

### 3.5. Other Enhancement Methods

The method of enhancing the fluorescence of green fluorescent protein (GFP) chromophore effectively detects small distances and is utilized for studying DNA and RNA interactions, providing high-resolution data and sensitivity to short distances. It serves as a valuable alternative to or combination with FRET (fluorescence resonance energy transfer). Fluorescence enhancement in synthetic GFP (green fluorescent protein) chromophore (GFPc) analogs can be achieved through two methods: physical encapsulation and chemical modification [225]. These approaches have great potential in enhancing the fluorescence of synthetic GFPc analogs, making them promising candidates for application in novel sensors or fluorescent probes. The fluorescent dye PicoGreen exhibits limited luminescence when in solution, but it forms a highly luminescent complex upon binding to dsDNA. During binding, it undergoes intercalation (insertion between DNA base pairs) and electrostatic interactions, which immobilize the dye molecule [226]. This immobilization leads to a significant fluorescence enhancement of PicoGreen, resulting in an increase of over 1000 fold compared to its free state. The fluorescence enhancement observed when selectively detecting potassium (K^+^) and cesium (Cs^+^) ions using bis-15-crown-5 and bis-18-crown-6 systems, respectively, has been termed as self-assembling fluorescence enhancement (SAFE) [227]. Self-assembled structures enhance fluorescence when interacting with specific targets, like barium ions. A bis-15-crown-5-naphthalenediimide compound acts as a chemosensor, inducing self-assembly and forming an intramolecular excimer, resulting in increased fluorescence. The fluorescence probes, RPd2 and RPd3, containing conjugated allylidene-hydrazone ligands, exhibit a 170-fold fluorescence enhancement and better selectivity for Pd^2+^ compared to other metal ions [228]. RPd2, with enhanced specificity and detectability, holds potential for Pd^2+^ analysis in contaminated water and soil. Adding N-phenyl substituents to 4-aminostilbenes in a study on various trans isomers of stilbene derivatives led to a more planar structure, resulting in a red shift in absorption and fluorescence spectra [229]. This enhanced fluorescence was attributed to the increased charge-transfer character in the excited state. The N-phenyl derivatives exhibited high fluorescence quantum yields and low photoisomerization quantum yields, indicating significant fluorescence enhancement. The binding of curcumin with α- and β-cyclodextrins (CDs) leads to a remarkable increase in its fluorescence, up to 7 times stronger when combined with 30 mM HP-β-CD [230]. This enhanced fluorescence could be valuable for fluorescence-based detection methods involving this important pharmaceutical compound. A novel type of carbon dots, co-doped with fluorine and nitrogen (F, N-doped CDs), shows improved fluorescence under UV light and high pressure (0.1 GPa). At regular atmospheric pressure (1.0 atm), F, N-doped CDs’ fluorescence intensity increases with UV light exposure (5 s to 30 min) and emits light of a different color (blue-shift) from 586 nm to 550 nm [231]. Another alternative method was reported by the synergic usage of dye-doped nanoparticles that encapsulated 200 dye molecules in a single 22 nm organosilicate particle [232] and high surface area porous films as biosensosing substrates. This method reported LOD to be as low as 21.3 fg/mL for detecting botulinum neurotoxin type A(BoTN/A) [233].

While plasmonic enhancements offer significant improvements in sensitivity and detection limits, several factors including quenching risks, cost and complexity, comparison with mainstream methods, reproducibility and scalability warrant careful consideration. While quenching is particularly useful in fluorescence sensors for detecting the presence of quencher molecules, plasmonic materials, particularly metal nanoparticles, can sometimes expedite the quenching process rather than enhancing fluorescence, especially when the fluorophore is too close to the metal surface. This proximity-dependent quenching can reduce the efficiency of fluorescence emission, undermining the enhancement effect and potentially leading to false negatives or less sensitive detection [167]. Implementing plasmonic materials in fluorescence-based detection systems can increase both the cost and complexity of the sensor design and fabrication. Plasmonic nanoparticles like gold and silver are more expensive than conventional fluorophores and may require sophisticated synthesis and functionalization techniques to ensure biocompatibility and stability [234]. Additionally, integrating these materials into devices necessitates precise control over nanoparticle size, shape, and spacing to achieve the desired plasmonic effect, further complicating the sensor design and increasing production costs [235]. While plasmonic enhancements can offer superior sensitivity and lower detection limits, the overall utility of these sensors must be evaluated in the context of existing technologies. Factors such as ease of use, durability, cost-effectiveness, and the ability to integrate into existing analytical workflows are crucial for determining whether plasmonic strategies offer a tangible advantage [236]. Achieving consistent and reproducible enhancements across different batches of plasmonic materials can be challenging, potentially limiting the scalability of these approaches for commercial applications. Variability in nanoparticle size, shape, and surface chemistry can lead to inconsistent fluorescence enhancement, affecting the reliability of sensor readings. The various enhancement types, the processes described, and the corresponding achieved enhancement factors are succinctly summarized in Table 1.

## 4. Diagnostics by Plasmonic Fluorescence Sensors

Plasmonic biosensors can be classified mainly into two types: one using thin metal-based films and the other employing inorganic plasmon resonant nanostructures [237,238]. One of the most common and well-known biosensors is “surface plasmon resonance” (SPR), which utilizes a metal-based film sensor, typically made of gold, for studying biomolecular interactions [239]. These biosensors provide a variety of sensing methods, and certain ones even integrate both platforms. Plasmon resonant nanostructures, whether utilized independently or in conjunction with film-based sensing, offer a wide range of sensing capabilities, making them valuable tools in biosensing applications [240].

Several widely used techniques for detecting infectious viruses include gene sequencing, cell culturing, immunofluorescence assays, hemagglutination assays, viral plaque assay, viral flow cytometry, ELISA, and nucleic acid amplification tests (NAAT) like polymerase chain reaction (PCR) and real-time quantitative PCR (RT-qPCR) [241,242,243,244,245,246]. While these methods have demonstrated successful results [237], they also have significant limitations that have hindered their use in future disease detection. The need for rapid and cost-effective diagnostic methods has driven the emphasis towards developing real-time biosensing platforms. In recent times, numerous plasmonics platforms have risen to the challenge of providing on-site approaches as supplements to conventional diagnostic techniques that rely on PCR and ELISA [247]. Over the past decades, various biosensor technologies have emerged for infectious disease detection, and among them, plasmonic applications have garnered considerable interest due to their versatility, label-free monitoring capability, and quick response times [248]. The diagnostics for viral and bacterial infections using plasmonic fluorescence sensors are comprehensively detailed in Table 2 for viruses and Table 3 for bacteria, respectively.

**Table 1:** Enhancement types, processes mentioned and achieved enhancement factor.

Enhancement Type	Process	Enhancement Factor *	References
MEF	SPCE	30 and 110 times compared to normal SPCE and free space emission respectively	[178]
MEF	Nanosphere lithography for 3D Au-NHDAs	Over 400 times	[182]
MEF	Plasmonic Antenna Arrays	$10^{4}$–$10^{5}$ times	[185]
MEF	TiO_2_ adhesion layer	25 times	[186]
MEF	Metallic Nanoparticles	Over 50 times	[173]
MEF	Plasmonic Metasurfaces	423 times	[249]
MEF	2-color DNA assay	1000 times	[176]
MEF	Plasmonic Nanodots	2970 on average and $4.5\times 10^{6}$ times at hotspots	[190]
PPCIFE	Combining Ag plasmon with PCs	Upto 150 times	[196]
PPCIFE	Combining AuNPs with SPCMs	20 times	[197]
PPCIFE	PCs coupled emission	1200 times	[198]
PPCIFE	Lattice Arrangement with Au film	40 times per molecule	[199]
PPCIFE	Combining plasmonic fluor NPs and PCs with a fluorescent dye	52 times	[200]
PPCIFE	Hybrid Ag and hydrophobic 3D PCs	87 times	[201]
PCs Based	CPCs coupling	1000 times	[207]
PCs Based	CoFe_2_O_4_@SiO_2_@Ag CPCs	12.6 and 17.6 times for RB and FITC, respectively	[209]
PCs Based	Sandwiched crystal using Al_2_O_3_ and TiO_2_	Upto 60 times	[210]
PCs Based	TiO_2_ in conjunction with 2D PCs	10 times	[211]
Hydrogel Based	Fructose exposure	10 times	[220]

* Enhancement Factor: a ratio of the fluorescence, which was enhanced by surface plasmons, to the fluorescence, which was measured on the surface without surface plasmon.

**Figure 4:**
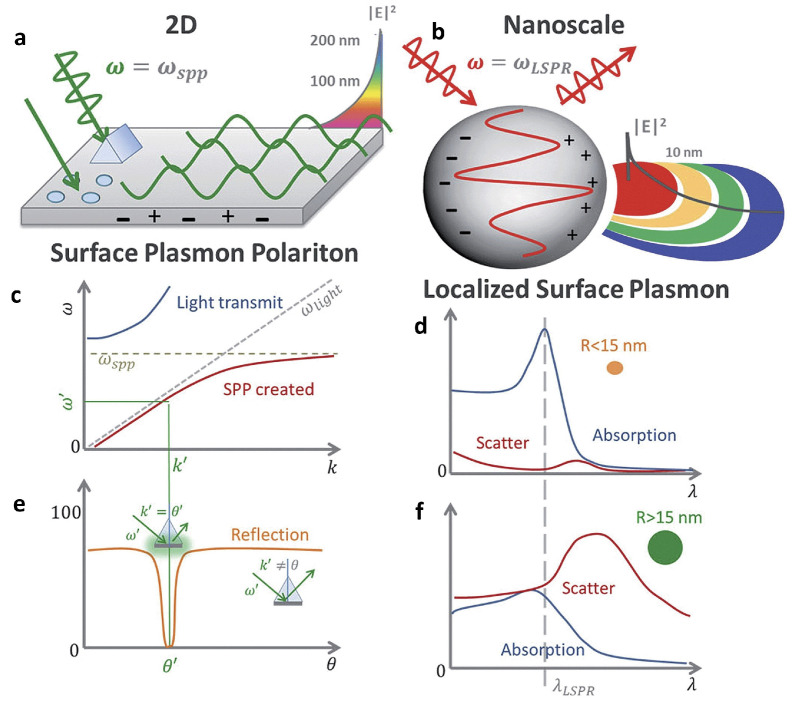
Surface and localized surface plasmon resonances. (**a**) On a 2D surface, electron oscillations create surface plasmon polaritons (SPPs) that couple with an electromagnetic field, propagating with reduced amplitude away from the interface. (**c**) SPPs are excited at specific wave vectors, leading to a field that decays exponentially from the surface. SPP resonance (**e**) is restricted to specific incident angles due to momentum matching. (**b**) LSPR arises when the metal nanoparticle is smaller than the incident wavelength, resulting in synchronized electron oscillations that lead to significant absorption, scattering capabilities and an intensified local electromagnetic field. In the case of small particles (less than 15 nm), (**d**) absorption is the predominant effect with a substantial absorption cross-section. Conversely, for larger nanoparticles (greater than 15 nm), (**f**) scattering becomes the dominant factor. Reproduced from ref. [168], Copyright (2015), with permission from the Royal Society of Chemistry.

The difference between SPPs and LSPR is determined by the dimension of the plasmonic nanomaterial [168]. For a thin film, surface oscillations result in the propagation of charge waves, SPPs, as shown in Figure 4a. The dispersion curve in Figure 4c indicates the light’s wave vector required to excite an SPP at a given interface. The angle at which a grating or prism can provide the necessary momentum to excite the SPP is determined by this dispersion curve, resulting in light absorption and a dip in the reflection or transmission spectrum as shown in Figure 4e. LSPR occurs when plasmonic nanomaterial’s dimensions are smaller than the incident light’s wavelength, Figure 4b. Changes in the LSPR peak position induced by the local environment can be easily detected using a standard UV-Visible spectrometer, eliminating the need for additional gratings or prisms, Figure 4d. Incident light can trigger LSPR, causing energy emission as absorption or scattering, determined by particle size; see Figure 4d,f.

### 4.1. Viral Infections

#### 4.1.1. COVID-19

By demonstrating exceptional performance in rapidly and accurately detecting SARS-CoV-2 RNA from human saliva and nasal specimens and achieving 100% sensitivity and specificity, even when dealing with distinct SARS-CoV-2 variants, rapid solution heating using plasmonic thermocycling is achieved [250]. This is accomplished through the excitation of nanoparticles using infrared light, enabling successful reverse-transcriptase qPCR (RT-qPCR) within a reaction vessel containing polymerase chain reaction chemistry, fluorescent probes, and plasmonic nanoparticles. Additionally, by using compact optical components, both thermocycling and multiplexed fluorescence monitoring are enabled, making the instrument suitable for point-of-care use. The demonstrated process achieved a sample-to-result time of 22–23 min, encompassing sample preparation, while achieving a LOD of 2.2–4.4 copies per microliter, which stood competitively alongside alternative approaches. The study in [251] introduces a method for the detection of SARS-CoV-2 antibodies in human serum and saliva and quantifies immunoglobulin avidities against various coronavirus antigens utilizing plasmonic gold substrates for near-infrared fluorescence enhancement. The nanostructured plasmonic gold (pGOLD) SARS-CoV-2 IgG/IgM assay demonstrates notable accuracy, achieving 100% sensitivity in identifying cases two weeks after COVID-19 symptoms onset and exhibiting a high specificity of 99.78%, based on 454 negative samples. The distinct pattern of detecting IgM before IgG, along with a significant proportion (87%) of cases showing IgM positivity six days after symptom onset, offers valuable information for assessing early responses to coronavirus infection.

Although fluorescence sensors offer a straightforward analytical approach, autofluorescence from biological samples can lead to inaccurate results because of the interferences. Fluorescence near-infrared (NIR) nanosensors, made from low-toxic materials to facilitate greener diagnostics, as introduced in [252], can lower background signals significantly. While still in its early stages, this research holds promise for sustainable SARS-CoV-2 detection. Reported in [253], a biosensor utilizing AuNPs achieves rapid and selective detection of COVID-19 viral RNA in 40 min by utilizing synthesized 17 nm AuNPs. These nanoparticles exhibit simultaneous colorimetric, surface-enhanced Raman scattering (SERS), and fluorescence signals due to their inherent aggregation behavior and affinity for diverse bio-molecules. The sensor achieves a limit of detection of femtomolar level in all triple modes, with 259 fM in fluorescence mode. The triple-mode signals of the sensor are verified with each other to enhance the accuracy of experimental results, and its ability to identify single-base mismatches in each mode minimizes false negative/positive readings of SARS-CoV-2, enabling rapid, sensitive, and selective detection of COVID-19.

#### 4.1.2. Influenza Virus

**Figure 5:**
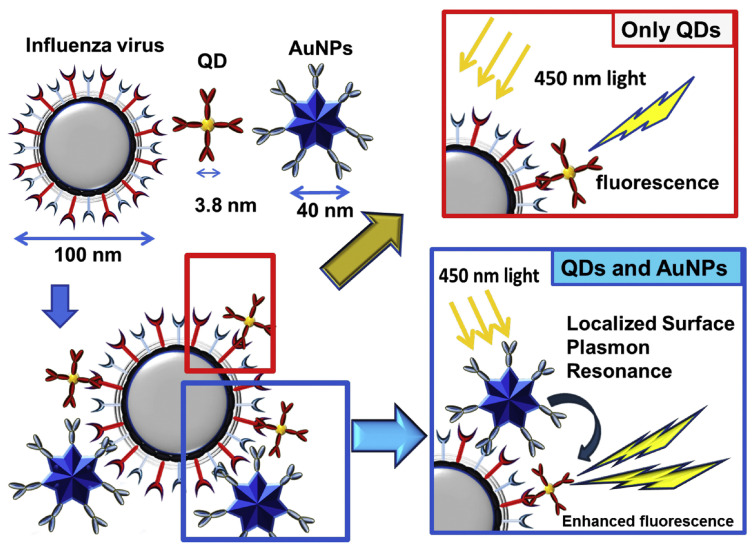
Schematic illustration of the detection mechanism of influenza virus using the LSPR-induced fluorescence nanobiosensor. Reprinted from ref. [255], Copyright (2016), with permission from Elsevier.

Researchers employed a localized surface plasmon-coupled fluorescence fiber-optic biosensor (LSPCF-FOB) for detecting swine-origin influenza A (H1N1) viruses (S-OIV) [254]. The LSPCF-FOB system demonstrated a lower limit of detection at 13.9 pg/mL for recombinant S-OIV H1 protein, surpassing conventional capture ELISA by $10^{3}$ fold using the same antibodies. For clinical S-OIV isolates, the LSPCF-FOB platform detected at $8.25\times 10^{4}$ copies/mL, while conventional capture ELISA only managed $2.06\times 10^{6}$ copies/mL. In addition, a highly sensitive immunofluorescence biosensor for rapid detection of H1N1 influenza virus is reported, which combines LSPR-induced optical transduction from AuNP-labeled anti-neuraminidase (NA) antibody (anti-NA Ab) with fluorescence signal amplification from adjacent alloyed CdSeTeS QD-labeled anti-hemagglutinin (HA) antibody (anti-HA Ab) [255]. As illustrated in Figure 5, the biosensor utilizes an antigen-antibody interaction, with LSPR enhancing the fluorescence of nearby alloyed QDs to detect the influenza virus, achieving detection limits of 0.03 pg/mL in deionized water and 0.4 pg/mL in human serum for H1N1 virus, and 10 PFU/mL for clinically isolated H3N2.

**Figure 6:**
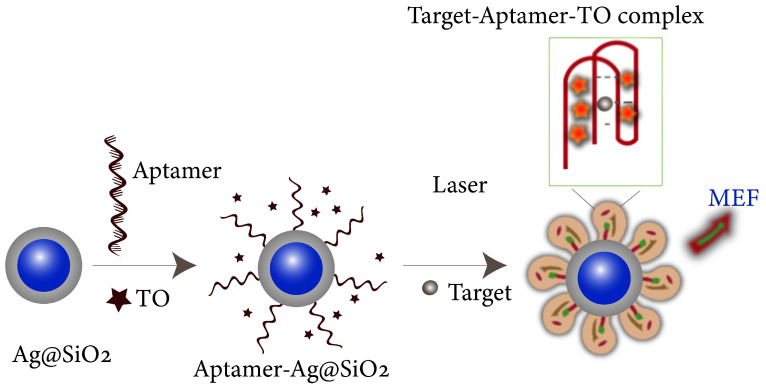
A schematic representation showing the preparation of the aptamer-Ag@SiO_2_ sensor and the process for detecting the rHA protein of H5N1. Reproduced from ref. [256], Copyright (2016), with permission from Elsevier.

A sensitive fluorescent aptasensor was developed for detecting recombinant hemagglutinin (rHA) protein of the H5N1 influenza virus in human serum [256,257]. It utilizes guanine-rich anti-rHA aptamers immobilized on Ag@SiO_2_ nanoparticles as a metal-enhanced fluorescence sensing platform with Thiazole orange (TO) as the fluorescent tag (Figure 6). In the presence of rHA protein, a stable G-quadruplex complex excites the TO fluorescence emission, achieving high sensitivity without covalent labeling and low background noise. The system can detect H5N1 rHA protein in aqueous buffer and human serum, with detection limits of 2 and 3.5 ng/mL, respectively, and completes the detection process within 30 min in a PE tube, making it suitable for point-of-care diagnosis.

#### 4.1.3. Human Immunodeficiency Virus (HIV) Infection

**Figure 7:**
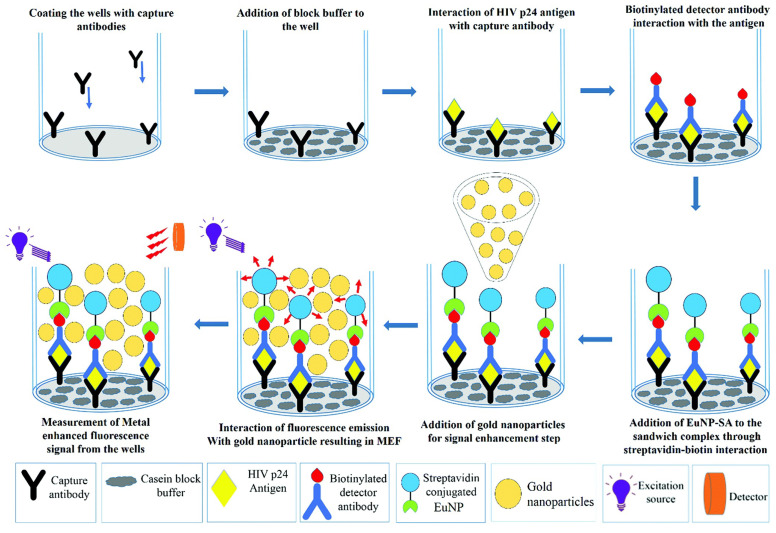
A schematic representation of the process involved in MEF based ENIA. Reprinted from ref. [259] under a CC-BY-NC 3.0 license, Copyright (2019), with permission from the Royal Society of Chemistry. Disclaimer: The licensor does not endorse you or your use. For the full license, please visit (accessed on 22 September 2023) https://creativecommons.org/licenses/by-nc/3.0/.

**Figure 8:**
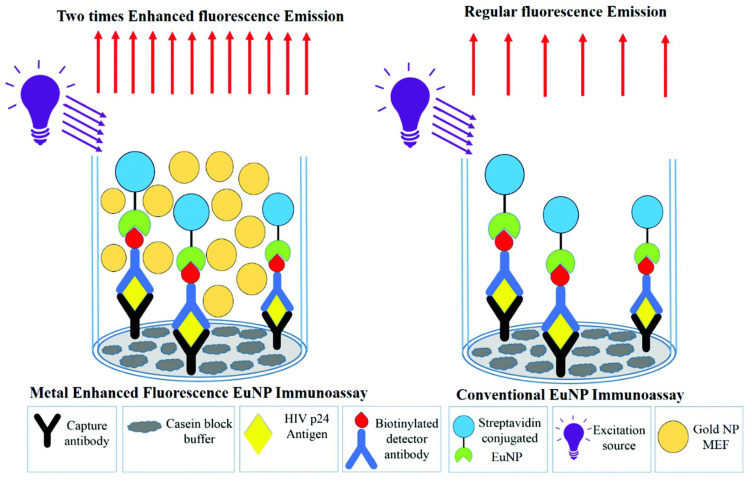
A schematic illustrating the contrast in fluorescence emissions between the conventional immunoassay and the MEF based ENIA. Reprinted from ref. [259] under a (CC-BY-NC) 3.0 license, Copyright (2019), with permission from the Royal Society of Chemistry. Disclaimer: The licensor does not endorse you or your use. For the full license, please visit (accessed on 21 September 2023) https://creativecommons.org/licenses/by-nc/3.0/.

A technique using single-molecule fluorescence has been developed to detect HIV DNA fragments without labels or enzymes [258]. The method utilizes a nucleic acid sensor anchored to a triangular gold nanoplate on one end and a guanine-rich hairpin structure, HP1, on the other, complemented by a hairpin probe, HP2. In the absence of target HIV DNA, this structure remains closed, producing a weak NMM fluorescence signal. When HIV DNA is present, the hairpins open to form numerous G-quadruplex structures, amplifying the fluorescence, which the plasmon resonance effect of the gold nanoplate further intensifies. This study introduces a novel application of Metal-Enhanced Fluorescence (MEF) in immunoassays, employing gold nanoparticles to enhance the existing europium nanoparticle-based immunoassay (ENIA) for detecting p24, an early biomarker of HIV infections [259]. Figure 7 provides a comprehensive protocol for the immunoassay, while Figure 8 illustrates the enhancement in signal strength following the introduction of gold nanoparticles. This modification significantly enhances signal strength, leading to a 10-fold improvement in the limit of detection for p24, which is 0.19 pg mL^−1^, far surpassing the conventional assay’s limit of 1.80 pg mL^−1^.

#### 4.1.4. Hepatitis

**Figure 9:**
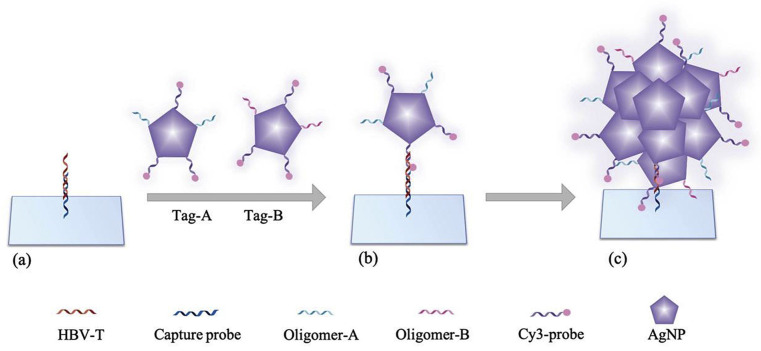
(**a**) Target DNA sequences are exposed to the microarray, where capture probes are immobilized. (**b**) The functionalized AgNPs (Tag-A and Tag-B) are introduced to the microarray and form hybrids with the adjacent regions of the target DNA sequences. (**c**) The formation of AgNP aggregates is triggered by the hybridization process. Reprinted from ref. [260], Copyright (2019), with permission from Elsevier.

**Figure 10:**
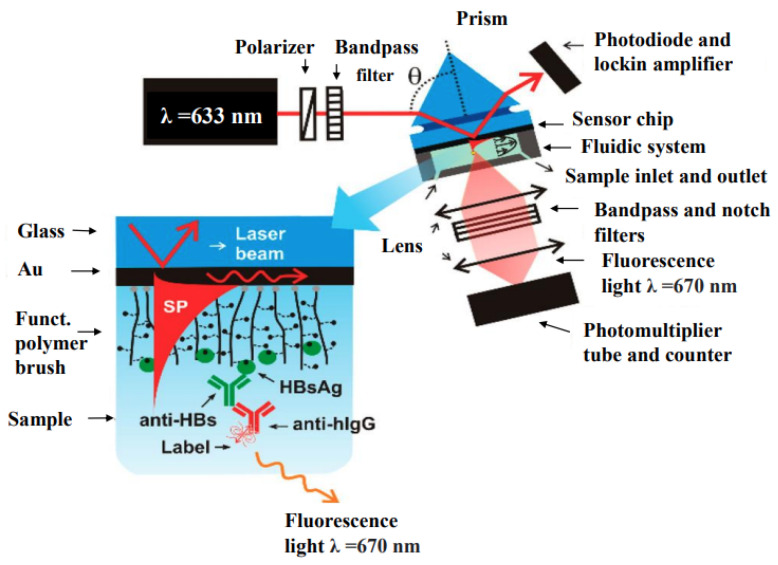
Illustrations showing a biosensor using plasmon-enhanced fluorescence spectroscopy, featuring an in-depth view of the sensor chip containing a binding matrix composed of poly(HPMA-*co*-CBMAA). Reprinted from ref. [261] under a (CC-BY) 4.0 International license, Copyright (2022), American Chemical Society. Disclaimer: The licensor does not endorse you or your use. For the full license, please visit (accessed on 15 November 2023) https://creativecommons.org/licenses/by-nc/4.0/.

**Figure 11:**
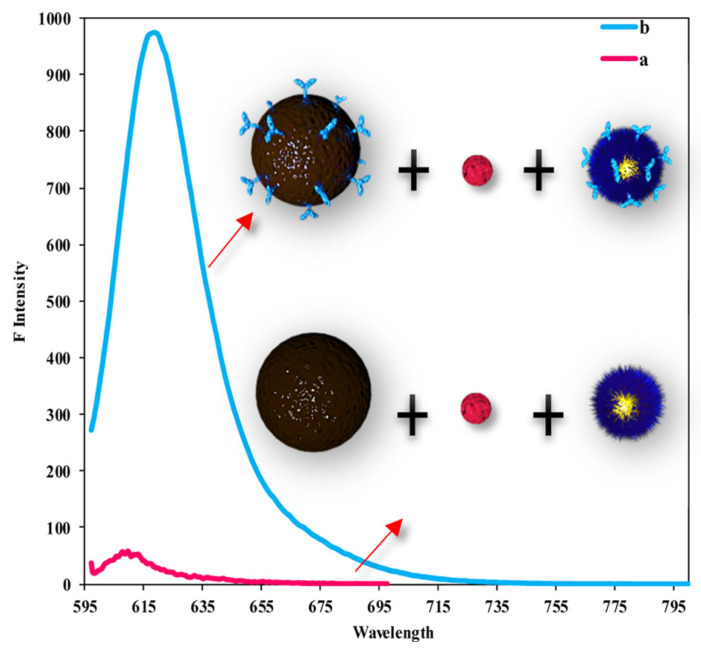
Impact of HBV-antibody (Ab) on the formation of the immunosensor: (a) measuring experiment and (b) Control experiment. Reprinted from ref. [262], Copyright (2019), American Chemical Society.

A fluorescence enhancement strategy was developed using silver nanoparticle (AgNP) aggregates for detecting hepatitis B virus (HBV) DNA sequences [260]. AgNPs were modified with recognition and hybrid probes as in Figure 9. When target DNA was present, it formed sandwich complexes with AgNPs, leading to aggregation and significant fluorescence enhancement. The method achieved a low detection limit of 50 fM, which is 1560 times better than un-enhanced assays, and demonstrated that there was a strong log-linear correlation between the concentrations of HBV DNA ranging from 100 fM to 10 nM and their corresponding fluorescent intensity. Additionally, the technique exhibited high specificity, detecting single-base mismatches and target DNA even in the presence of genomic DNA interference. This approach holds potential for high-throughput disease diagnosis beyond HBV detection. The biosensor in [261] used a plasmonically amplified fluorescence sandwich immunoassay to detect minute amounts of hepatitis B antibodies in clinical saliva, offering noninvasive sample collection. To ensure specific detection, the researchers prevented nonspecific adsorption of biomolecules in saliva by using specific brushes of poly[(N-(2-hydroxypropyl) methacrylamide)-*co*-(carboxybetaine methacrylamide)] (poly- [HPMA-*co*-CBMAA]) on the gold sensor surface, modified with hepatitis B surface antigen as shown in Figure 10. The biosensor’s results were validated against ELISA responses measured using serum samples from the same patients in a certified laboratory. It showed excellent resistance to fouling from saliva samples and accurately distinguishing between highly positive clinical saliva samples (with respective serum ELISA response > 1 IU/mL) and negative clinical saliva samples (with respective serum ELISA response < 0.01 IU/mL). A novel magnetoimmunoassay was developed to detect hepatitis B virus surface antigen (HBsAg) using a new fluorescence label formed by covalently conjugating thionine—gold nanoparticles (GNP-Th) that enhances its intensity via gold nanoparticles’ plasmonic effect [262]. The immunosensor utilizes magnetic nanoparticles (MNP-Ab) to capture GNP-Th once the sandwich probe forms in the presence of HBsAg. Subsequently, the proteolytic enzyme facilitates the release of GNP-Th into the solution by digesting the grafted antibodies on the GNP-thionine hybrid, leading to the release of the amplified fluorescent labels. The effectiveness of the proposed approach was assessed by comparing the signal responses in both control and measuring experiments. As illustrated in Figure 11, a significant disparity in fluorescence intensity is evident between the control experiment (**a**) and the measurement experiment (**b**). The method is simple, cost-effective, and exhibits high sensitivity and selectivity, detecting HBsAg in a wide range from $4.6\times 10^{-9}$ to 0.012 ng/mL with a low detection limit of $4.6\times 10^{-9}$ ng/mL.

#### 4.1.5. Ebola

**Figure 12:**
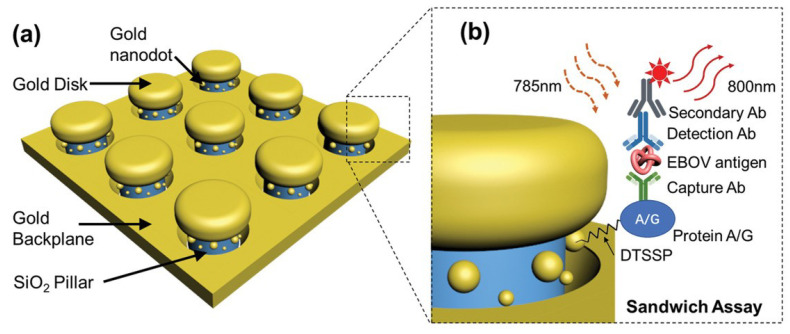
Nanostructures and fluorescence sensing techniques for the EBOV sensor. (**a**) Nanoantenna array illustration. (**b**) Detailed view highlighting the features of individual nanopillar structures and an example of an on-chip EBOV sandwich assay configuration. Reprinted from ref. [263], Copyright (2019), with permission from WILEY-VCH Verlag GmbH & Co. KGaA, Weinheim, Germany.

Developed as an on-chip immunoassay platform for highly sensitive Ebola virus (EBOV) antigen detection, the 3D plasmonic nanoantenna assay sensor showcases substantial enhancement in fluorescence intensity during immunoassays, particularly in comparison to flat gold substrates [263]. The nanoantenna array sensor platform includes a plasmonic nanoantenna array with EBOV capture agents attached to its surface through a molecular linker layer Figure 12a. The nanoantenna array is composed of silicon dioxide nanopillars, each featuring an Au nanodisk on top, Au nanodots on the sidewalls, and an Au backplane at the pillar base.

Figure 12b provides an exploded view illustrating the concept of an EBOV sandwich assay on this nanoantenna array platform. Using the nanoantenna biosensor, EBOV soluble glycoprotein (sGP) was successfully detected in human plasma at an impressive concentration of $220\;\mathrm{fg}\;{\mathrm{mL}}^{-1}$, a sensitivity enhancement of 240,000 times compared to the current EBOV immunoassay’s $53\;\mathrm{ng}\;{\mathrm{mL}}^{-1}$ antigen detection limit. The sensor’s identification of sGP-spiked plasma samples at double the detection limit with 95.8% sensitivity in a simulated clinical trial demonstrates its exceptional capacity for highly sensitive EBOV antigen detection, outperforming existing methods and holding promise for advanced disease diagnosis and pathogen detection in public health.

#### 4.1.6. Dengue

**Figure 13:**
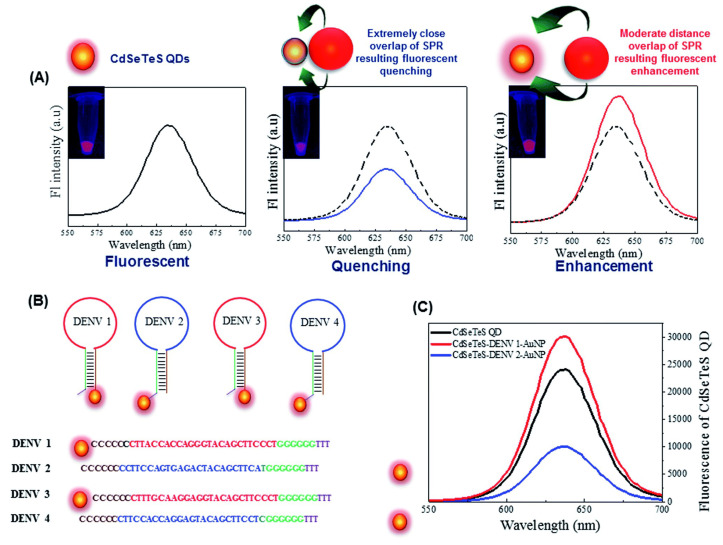
(**A**) Schematic representation of the distance-based LSPR effect of AuNPs on CdSeTeS QDs. (**B**) Schematic of four hairpin probes for sensing purposes. (**C**) Fluorescent characteristics of CdSeTeS QDs and CdSeTeS QDs–dsDNA–AuNP nanocomposites with DENV 1 and DENV 2. Reprinted from ref. [264] under a (CC-BY-NC) 3.0 license, Copyright (2019), with permission from the Royal Society of Chemistry. Disclaimer: The licensor does not endorse you or your use. For the full license, please visit (accessed on 15 November 2023) https://creativecommons.org/licenses/by-nc/3.0/.

Optimizing the interaction between cadmium selenide tellurium sulphide fluorescent quantum dots (CdSeTeS QDs) and gold nanoparticles (AuNPs) has resulted in the development of a rapid and precise biosensor tailored for detecting dengue viruses (DENVs) serotypes 1 to 4 [264]. The innovation includes creating unique nanoprobes with serotype-specific hairpin single-stranded DNA molecules attached at different positions to CdSeTeS QDs, resulting in distinct fluorescence signals for each DENV serotype (Figure 13). The biosensor effectively detected target virus DNA in concentrations from $10^{-15}$ to $10^{-10}$ M using a fourplex reaction with AuNPs and nanoprobes, establishing femtomolar range detection for all serotypes, 24.6, 11.4, 39.8 and 39.7 fM for DENVs 1, 2, 3 and 4, respectively. The sensor’s versatility was highlighted by identifying and quantifying serotypes in complex RNA samples from DENV culture fluids, demonstrating its potential as a point-of-care diagnostic tool. In the pursuit of creating probes for precise identification of different dengue virus strains, gold nanoparticles (AuNPs) are combined with glutathione (GSH)-functionalized CdSe/ZnSeS core/alloyed shell quantum dots (Qdots), enhancing the photoluminescence quantum yield (ranging from 23% to 99%), representing an approximately 2–8-fold increase over that of the binary CdSe core [265]. The biosensor has successfully detected and distinguished DENV1–4 nucleic acids with high sensitivity, achieving detection limits ranging from 31 to 260 copies per mL. Comparing the sensitivity of a Qdot–molecular beacon (MB) to that of the AuNP–Qdot–MB demonstrated that the signal resulting from localized surface plasmon resonance in the AuNPs effectively amplified the fluorescence intensity of the Qdots, leading to an improved biosensor performance. Immunoglobulin-M (IgM)-based assays are of great value in detecting the initial phases of infectious diseases such as dengue and Zika [266,267]. In this research, the surface-enhanced fluorescence (SEF) technique was employed to develop specialized tags constructed using shell-isolated nanoparticles (Au-SHINs), which consist of a 100 nm core and a 10 nm silica shell, for detecting Immunoglobulin-M (IgM), a key factor in disease diagnosis. These particles were coated with a thin layer of the bright material Nile blue (NB), followed by an additional 5 nm layer of silica. These tags effectively detected IgM, even at low concentrations of just $10\;\mathrm{ng}\;{\mathrm{mL}}^{-1}$. In [268], a method using gold nanoparticles (AuNP) and nitrogen and sulfur codoped graphene quantum dots (N,S-GQDs) identifies various dengue virus (DENV) types and measures their DNA. This N,S-GQDs@AuNP composite detects DENV via fluorescence with dye-labeled DNA probes, and was validated by confocal microscopy. The DNA amount is quantified using differential pulse voltammetry (DPV) with methylene blue as a redox indicator. Findings show N,S-GQDs@AuNP effectively identifying individual DENV types at concentrations from $10^{-14}$ to $10^{-6}$ M, with a 9.4fM limit of detection. The sensor’s DENV serotype identification and quantification ability was confirmed with clinically isolated DENV DNA.

### 4.2. Bacterial Infections

#### 4.2.1. Tuberculosis (TB)

**Figure 14:**
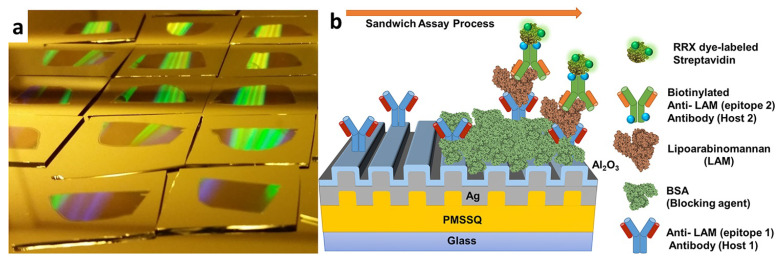
(**a**) A photographic representation of PMSSQ gratings created on glass slides measuring 1 inch by 1 inch, and (**b**) an illustration outlining the FLISA setup on the gratings, demonstrating the antibody sandwich structure used for detecting LAM. Reprinted from ref. [126], Copyright (2019). Disclaimer: The licensor does not endorse you or your use. For the full license, please visit (accessed on 1 December 2023) https://creativecommons.org/licenses/by-nc/4.0/.

Wood et al. introduced a highly sensitive technique to detect a tuberculosis biomarker, lipoarabinomannan (LAM), using plasmonic grating biosensors in clinical samples from HIV-negative tuberculosis patients [126]. They used cost-effective plasmonic gratings (Figure 14) to analyze LAM in urine samples through a single molecule fluorescence assay (FLISA), comparing it to other established testing methods. The findings demonstrated that the plasmonic grating-based single molecule FLISA accurately quantified tuberculosis LAM in complex urine samples from high-endemic areas, with minimal interference from urine composition. By setting a limit of detection at 1fg/mL, the method aligned well with tests in 19 out of 20 patients. A cost-effective method was developed to create a plasmonic grating, enhancing light-coupling efficiency in fluorescence-based sensors through a simple micro-contact printing process [269]. Rhodamine 6G (R6G) fluorescence on these gratings improved by 239 fold compared to plain glass. The platform was optimized for Interferon-gamma (IFNγ) detection, a common biomarker for M. tuberculosis infection and autoimmune diseases, using an immunofluorescence assay, achieving an impressive 500fg/mL limit of detection, outperforming that of ELISA, 2pg/mL. Simulations supported the fluorescence increase, reinforcing the experimental results.

#### 4.2.2. Cholera

A highly sensitive DNA-based fluorescent assay was developed for detecting the Vibrio cholera O1 OmpW gene linked to bacterial virulence, utilizing gold nanoparticles (AuNPs) and magnetic nanoparticles (MNPs) [270]. MNPs were modified with DNA probe 1 to target one end of the DNA sequence, while AuNPs were equipped with DNA FAM-probe 2 to target the other end. Upon hybridization of these probes with the target DNA, a sandwich structure was formed, which could be separated from non-hybridized materials using a magnetic field. By employing Dithiothreitol (DTT), the sandwich structures were disintegrated, releasing the probes, and the resulting fluorescence emitted by the released FAM-probe 2 on AuNPs was detected through fluorescence spectrophotometry, showcasing outstanding sensitivity with a LOD of 2.34 ng mL^−1^.

#### 4.2.3. Urinary Tract Infections (UTIs)

**Figure 15:**
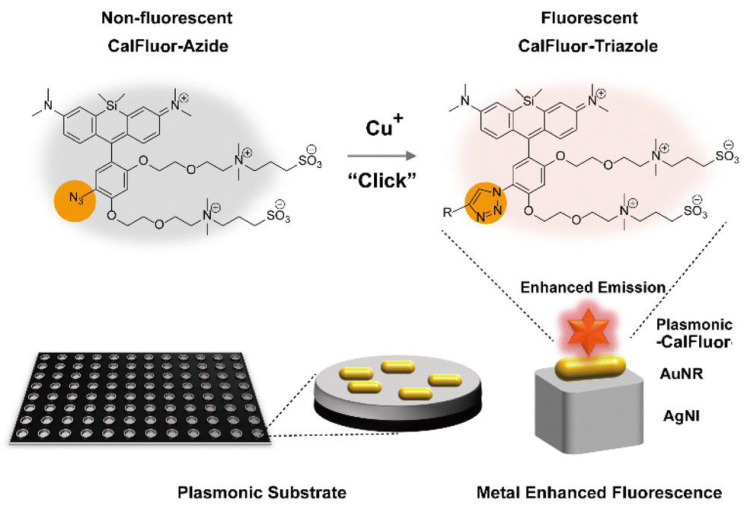
Principle of the chemical sensor employing cascade signal amplification for the quantification of Cu^2+^. Reprinted from ref. [271], Copyright (2023), with permission from the Royal Society of Chemistry.

The study in [271] presents an innovative chemical sensor using a cascade signal amplification strategy based on the Cu-catalyzed click reaction (Figure 15). Coupled with gold nanorods and silver nanoislands for enhanced fluorescence, the sensor can detect Cu^2+^ as low as 3.87 nM within a rapid 10-min timeframe. A demonstration of the proposed sensor’s utility involves conducting phenotypic antibiotic susceptibility testing (AST) on urinary tract infection (UTI) samples, achieved through indirect Cu^2+^ detection. Leveraging *E. coli*’s efficient Cu^2+^ adsorption, the sensor exhibits promise in detecting *E. coli* concentrations aligning with the gold standard, confirming its accuracy. Furthermore, exposing the bacteria to antibiotics for just 45 min resulted in a notably larger difference in intensity between the treated and control groups for ceftriaxone (Cef)-susceptible *E. coli* compared to ceftriaxone-resistant *E. coli*.

**Table 2:** Diagnostics of viral infections by plasmonic fluorescence sensors.

Disease	Analyte	Detection Method	LOD	References
COVID-19	SARS-CoV-2 RNA	Multiplexed qPCR (RT-qPCR)	(2.2–4.4) copies per μL	[250]
COVID-19	SARS-CoV-2 RNA	Triple-mode biosensing	259 fM	[253]
Influenza Virus	Influenza A (H1N1)	localized surface plasmon coupled fluorescence	13.9 pg/mL	[254]
Influenza Virus	Influenza A (H1N1)	LSPR combined with fluorescence signal amplification	0.03 pg/mL in DI water, 0.4 pg/mL in human serum	[255]
Influenza Virus	Influenza A (H3N2)	LSPR combined with fluorescence signal amplification	10 PFU/mL	[255]
Influenza Virus	Influenza A (H5N1)	MEF	2 ng/mL in aqueous buffer, 3.5 ng/mL in human serum	[256]
HIV	HIV DNA	Single-molecule fluorescence	0.83 fM	[258]
HIV	HIV-1 p24	MEF	0.19 pg mL^−1^	[259]
Hepatitis	HBV DNA	Fluorescence microarray	50 fM	[260]
Hepatitis	HBsAg	Magneto-immunoassay	4.6 $\times 10^{-9}\mathrm{ng}/\mathrm{mL}$	[262]
Ebola	EBOV	On-chip immunoassay	220 fg mL^−1^	[263]
Dengue	DENVs (1–4)	LSPR	24.6, 11.4, 39.8 and 39.7 fM for DENVs 1, 2, 3 and 4	[264]
Dengue	DENVs (1–4)	LSPR	31 to 260 copies per mL	[265]
Dengue	IgM	SEF	10 ng mL^−1^	[266]
Dengue	DENVs (1–4)	N,S-GQDs@AuNP nanoassembly	9.4 fM	[268]

**Table 3:** Diagnostics of bacterial infections by plasmonic fluorescence sensors.

Disease	Analyte	Detection Method	LOD	References
Tuberculosis	LAM	FLISA	1 fg mL^−1^	[126]
Tuberculosis	IFNγ	Immunofluorescence assay	500 fg mL^−1^	[269]
Cholera	O1 OmpW gene	Fluorescent assay	2.34 ng mL^−1^	[270]
UTIs	*E. coli*	Cu-catalyzed click reaction	3.87 nM	[271]

## 5. Perspective and Future Directions

This study aims to provide an insightful exploration of diagnostic strategies tailored to address viral and bacterial infections. The proposed techniques demonstrate a distinctive capability for detecting infections at lower concentrations compared to traditional diagnostic methods, offering a promising advancement in disease identification. A significant highlight of this research involves the utilization of plasmonic fluorescent sensors, which exhibit several key advantages. Notably, they showcase heightened sensitivity, allowing for the detection of even minute traces of pathogens in clinical samples. This sensitivity is complemented by an impressive level of selectivity, ensuring accurate differentiation between various infectious agents. Moreover, the inherent cost-effectiveness of plasmonic sensors presents an encouraging avenue for more accessible and widespread disease diagnostics. While plasmonic-based fluorescence biosensors offer promising attributes, it is evident that further investigation is needed. To the best of our knowledge, there is no diagnostic tool for deadly infectious diseases like gonorrhea based on plasmonic-based fluorescence, yet given the higher sensitivity of these biosensors, they could offer a valuable approach for diagnosis and, consequently, treatment improvement. A deeper exploration into their practical applications and the optimization of their performance parameters could amplify their potential impact on healthcare services. As these sensors show promise in significantly expediting disease detection and diagnosis, they could emerge as a pivotal tool for timely medical intervention.

## Data Availability

Data are contained within the article.
